# Hazard Perception and Prediction test for walking, riding a bike and driving a car: “Understanding of the global traffic situation”

**DOI:** 10.1371/journal.pone.0238605

**Published:** 2020-10-16

**Authors:** Candida Castro, Ismael Muela, Pablo Doncel, Pedro García-Fernández

**Affiliations:** 1 CIMCYC, Mind, Brain and Behaviour Research Centre, Faculty of Psychology, University of Granada, Granada, Spain; 2 Electronics and Computer Sciences Department, Faculty of Sciences, University of Granada, Granada, Spain; Tongii University, CHINA

## Abstract

To “put oneself in the place of other road users” may improve understanding of the global traffic situation. It should be useful enabling drivers to anticipate and detect obstacles in time to prevent accidents to other road users, especially those most vulnerable. We created a pioneering Hazard Perception and Prediction test to explore this skill in different road users (pedestrians, cyclists and drivers), with videos recorded in naturalistic scenarios: walking, riding a bicycle and driving a car. There were 79 participants (30 pedestrians, 14 cyclists, 13 novice drivers and 22 experienced drivers). Sixty videos of hazardous traffic situations were presented, divided into 2 blocks of 30 videos each: 10 walking, 10 riding a bicycle, 10 driving a car. In each situation presented, we evaluated the performance of the participants carrying out the task of predicting the hazard and estimating the risk. In the second block, after they had carried out the task, we gave them feedback on their performance and let them see the whole video (i.e., checking what happened next). The results showed that the holistic test had acceptable psychometric properties (Cronbach’s alpha = .846). The test was able to discriminate between the different conditions manipulated: a) between traffic hazards recorded from different perspectives: walking, riding a bicycle and driving a car; b) between participants with different user profiles: pedestrians, cyclists and drivers; c) between the two test blocks: the first evaluation only and the second combining evaluation with this complex intervention. We found modal bias effects in both Hazard Perception and Prediction; and in Risk Estimation.

## Introduction

To put themselves in the place of other users (as actor-observers) could enrich their situation awareness and improve their ability to perceive hazards (i.e. identify, recognise and react to potentially dangerous traffic situations), which correlates with the avoidance of accidents [1]. A “holistic picture of the traffic environment, comprehending the significance of objects and events” [2, p.37] may help the driver and other road users to understand how others act and predict their behaviour. Accident rates continue to cause concern and no effort should be spared by governments and research centres in trying to reduce the needless death and disability provoked by road accidents (i.e. 23,926 fatalities in 2016 in Europe, [3]). The distribution of fatalities by mode of transport was as follows: car or taxi 47%, pedestrian 22%, motorcycle 14%, bicycle 8%, lorry 5%, moped 3%. Globally, accident rates are even higher and affect vulnerable users to a greater extent, with pedestrians and cyclists representing 26% and two- or three-wheeled vehicles 28% of deaths. Car occupants account for 29%, with another 17% not identified [4]. Drivers’ HP may impact the crash injury severity significantly [5–7]. According to [8. Page 425]: “Hazard Perception (HP) testing and training appear to have the capability to reduce crash risk, (i.e the inclusion of a hazard-perception test in the UK driver licensing process has been estimated to reduce drivers’ non-low-speed public-road crash rates by 11.3% in the year following their test)”.

The literature includes studies that have explored what the HP skills of different road users are like from one unique traffic perspective. The majority of these studies have focused on analysing and improving the HP performance of car drivers [9–13]. The traditional HP test is used to evaluate to what extent the skill of perceiving traffic hazards has been developed [14]. The HP test of all drivers and riders is part of theory test to obtain a driving licence, e.g. in the UK (DVSA, *Driver & Vehicle Standards Agency*, DoT *Department for Transport*, 2020). This HP test measures the reaction time of participants facing different hazards on the road. The HP and Hazard Prediction Test (What Happens Next?), which measures the Situation Awareness (SA) of the driver, was subsequently created [2]. This too presents videos recorded from real driving situations in which, at the moment the video is cut, a “potential hazard” begins to unfold (“developing hazard”). The ability to identify, understand and project what will happen next in this traffic situation is evaluated using a series of questions: What is the hazard? Where is it? What will happen next? [10–19]. In this case, the precision of the participants is measured.

On the other hand, risk estimation can be measured with the question: To what extent does the situation you have seen appear dangerous? Respond 1 if it seems slightly dangerous, 6 if it seems highly dangerous [20]. Previous studies have concluded that young drivers are particularly susceptible to “optimism bias”, defined by Weinstein (1980) as the tendency to believe that one is more skilled and less likely to experience a negative event than one’s peers, and higher perceptions of driving skill are associated with lower perceptions of accident risk. [20–24]. found that novice drivers scored significantly lower than experienced drivers when they estimated their driving skills, such as driving ability, awareness of others and self-confidence in driving, although at the same time, they estimated traffic situations as more dangerous. For this reason they could be considered naive drivers. By contrast, reoffender drivers overestimate their skills and underestimate the dangers of traffic situations, which means they could be classified as “bold drivers”.

Although not so prolifically, HP has also been studied from the different perspectives of other road users. For example, the HP performance of pedestrians has also been analysed (in Israel: [25–27]). Other HP tests have analysed the HP skills of motorcyclists without driving experience [28] and in Australia the HP of car drivers and motorcyclists has been compared [29]. HP tests were also designed from the perspective of motorists in the UK [30, 31] and in other countries such as Italy [32, 33]. Recently, HP from the perspective of professional drivers has been studied. For instance, [34] analysed the HP of fire-engine drivers. Other tests that analysed HP from the perspective of cyclists (children, young people and adults) have been developed in Holland [35–38]. However, no HP test yet exists that combines the evaluation and/or training of road users from different perspectives.

Many make the assumption that the “not having seen” a pedestrian or cyclist means that the pedestrian or bike was difficult to see (it had little visual salience) because it was smaller or had only one headlight, especially if they were in busy or complex traffic situations [39]. However, expectations can be influenced by the frequency with which we interact with each type of road user [40]. In addition, according to [41] as the number of pedestrians or cyclists in a city increases, so the rates of victims begin to diminish in proportion. They are psychological effects that produce a diversion in mental processing, a distortion, an inexact judgment, an illogical interpretation, or what in general terms is called irrationality. In addition social biases affect our everyday interactions and our decision-making. They are seen to be influenced by the probability with which certain events occur; they generate in us expectations that influence us in our processing of information, in guiding our new perceptions or our visual searches, in the interpretation of situations (SA) and in decision-making [42]. In addition, drivers take decisions bearing in mind exclusively their own individual rather than the collective benefit [43].

If the usefulness of this holistic training could be demonstrated, it would mitigate to a considerable extent the difficulties in detection of vulnerable users [39] and reduce other road accidents. This study proposed a pioneering objective: To create a new holistic evaluation test of the skills of different road users (pedestrians, cyclists and drivers) in HP and Prediction and Risk Estimation, with dangers recorded from the different perspectives of road use (walking, riding a bicycle, in cars), in order to predict a profile of the safe user. And register distinct simple empirical variables: A) Hazard Detection Accuracy, B). HP and Prediction in Situation Awareness (Perception, Comprehension and Projection of the future situation) [2]. C). Risk Estimation. Secondly, to analyse the utility of a change of perspective, not only as a form of evaluation, but also to explore the influence of training in the skill of HP and Prediction on different road users.

## Method

### Participants

Seventy-nine participants (35 female and 44 male) took part in this experiment. The sample was divided into four groups according to their road user profile: A) 30 pedestrians (37.9% of the sample), aged between 15 and 57 (*M* = 21.87, *SD* = 7.637), none of them with a driving license (for any type of motor vehicle). B) 14 cyclists (17.7%), this being understood as people who usually use a bicycle as a means of transport to move from one place to another. The aforementioned cyclists were between 19 and 31 years old (*M* = 21.71, *SD* = 3.099) and held a car driving license (driving less than 5,000 Km/year). C) 13 novice drivers (16.5%), aged between 18 and 29 (*M* = 21.54, *SD* = 3.711), all of them in possession of a driving license. They could either have less than two years’ driving experience or more than two years but driving infrequently (less than twice a week) and driving less than 5,000 km per year. D) 22 experienced drivers (27.8%), aged between 19 and 60 (*M* = 39.18, *SD* = 12.894), who had more than two years’ driving experience, using the car frequently, and driving more than 10,000 km per year, composed the fourth group. This manuscript reports results of N = 79 participants. They are posted on the Open Science Framework at https://osf.io/xcz6r/ The sociodemographic characteristics of the study sample are shown in Table 1.

**Table 1:** Sociodemographic information. Breakdown of participants’ sociodemographic information (age, gender years with driving licence, driving frequency in the last 12 months, accidents in the last 12 months and kilometres driven last 12 months) per group (pedestrians, cyclists, novice drivers and experienced drivers).

	***Pedestrians***						***Cyclists***					
	*N*	*Mean*	*Median*	*Min*	*Max*	*SD*	*N*	*Mean*	*Median*	*Min*	*Max*	*SD*
**Age**	30	21.87	20	15	57	7.64	14	21.71	20.5	19	31	3.099
**Gender^a^ + Gender Percentage**	15/15		0.5	0 = M 50%	1 = F 50%		10/4		0	0 = M 71.4%	1 = F 28.6%	
**Years with licence**							14	2.64	2	0	37	8.38
**Driving frequency in the last 12 months**^**b**^							14	0,29	0	0	1	0.469
**Accidents in the last 12 months**^**c**^							14	0,14	0	0	2	0.535
**Kilometres driven last 12 months**							14	948.6	225	0	8000	2103
	***Novice Drivers***						***Experienced drivers***					
	*N*	*Mean*	*Median*	*Min*	*Max*	*SD*	*N*	*Mean*	*Median*	*Min*	*Max*	*SD*
**Age**	13	21.54	20	18	29	3.71	22	39.18	37.5	19	60	12.89
**Gender^a^ + Gender Percentage**	7/6		0	0 = M 53.8%	1 = F 46.2%		12/10		0	0 = M 54.5%	1 = F 45.5%	
**Years with licence**	13	1.77	2	0	6	1.42	22	16.55	16	2	38	9.4
**Driving frequency in the last 12 months**^**b**^	13	0.23	0	0	1	0.44	22	1	1	1	1	0
**Accidents in the last 12 months**^**c**^	13	0.23	0	0	1	0.44	22	0.09	0	0	1	0.294
**Kilometres driven last 12 months**	13	1902	500	20	8000	2716	22	31455	20000	10000	100000	25357

*Median Value reported

a) 0 = Male, 1 = Female

b) 0 = Once or less than once per week, 1 = Twice per week. (Average reported)

c) 0 = 0, 1 = 1, 2 = 2 or more

All participants had normal or correct-to-normal vision. They were recruited from: A.) Different departments of the University of Granada (UGR), students and staff, and B.) Different driving schools at Granada (Autoescuela Genil, Ogíjares, Victoria and Luna). These participants were attending different training courses (i.e., first-aid courses). They received a T-shirt from the UGR as compensation. Ethical principles in the declaration of Helsinki for research involving human participants were followed in the current study. Permission was obtained from the Ethics Committee for Research with Humans 327/CEIH/2017: “HP and Prediction Test for vulnerable users: pedestrians, cyclists and motorists, using a change of perspective as evaluation and training”. We declare that we have no known competing financial interests or personal relationships that could have appeared to influence the work reported in this paper.

A (3)x(2)x4 mixed factorial design was employed (3) driving perspectives (walking, riding a bicycle and driving) X (2) Blocks (B1, no training and B2, training) X 4 road user profile (pedestrian, cyclist, novice and experienced driver).

Repeated Measures were taken from the traffic perspective (walking, riding a bicycle and driving) and the effect of training (B1, no training and B2, training block). The independent variable measured between groups was the type of road user (pedestrian, cyclist, novice and experienced driver).

Three dependent variables were measured: Q1. Hazard Detection, Hits (Did you see the hazard?), Q2. Situation Awareness (What might happen next?) and Q3. Subjective Risk Estimation with ratings from 1 to 6 (How dangerous does this situation seem to you?).

## Materials

**Fig 1:**
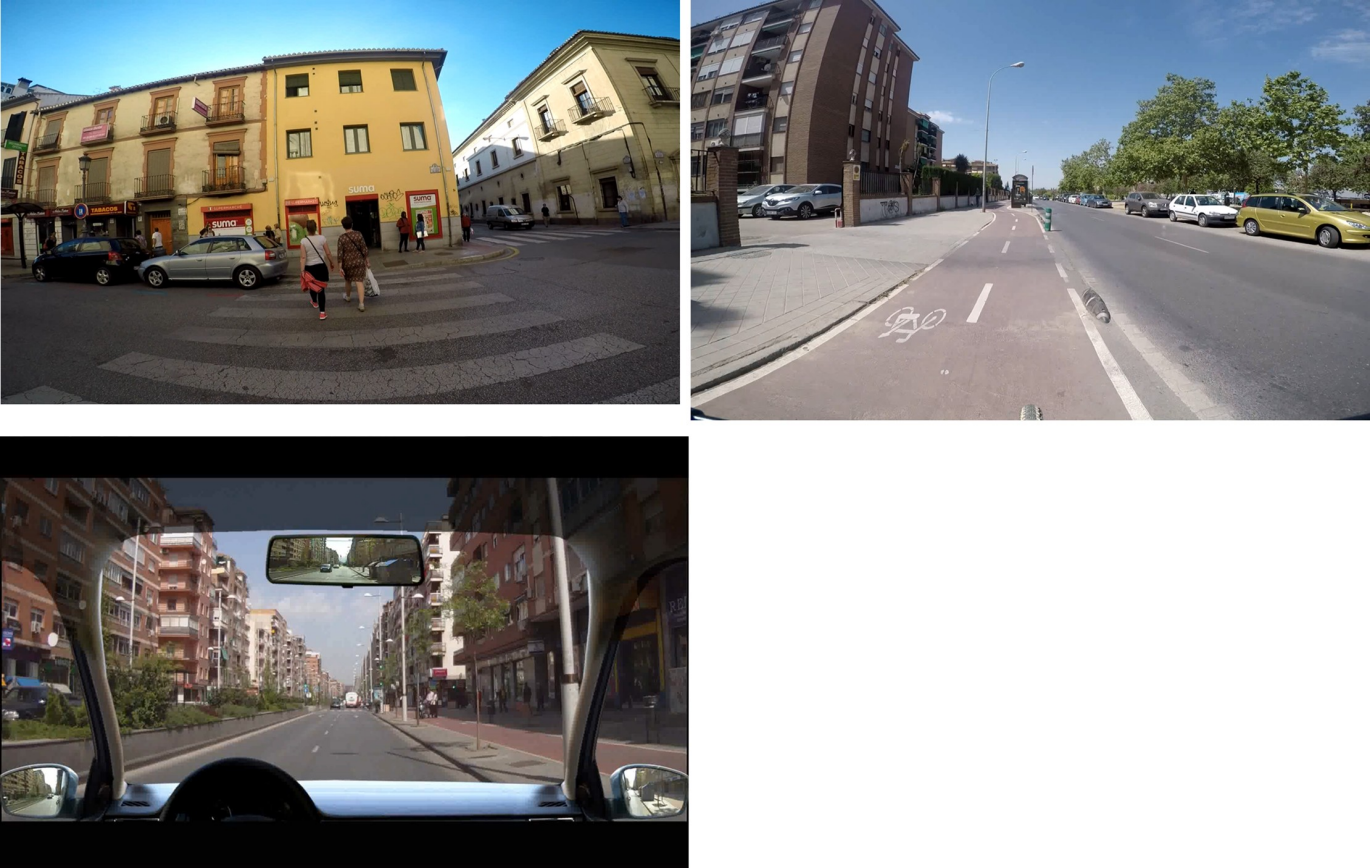
**A.** Example of the clips recorded from the perspective of walking. **B.** Example of the clips recorded from the perspective of riding a bicycle. **C.** Example of the clips recorded from the perspective of driving a car.

With the objective of creating this Holistic HP and Prediction test, video clips were recorded around the province of Granada (Spain) from three different perspectives of the traffic environment: walking, riding a bicycle and driving (See Fig 1A–1C).

More than 200 videos were recorded and compose our database. The videos’ resolution was 1080 p, 50 frames/second and the screen resolution 1920 x 1080. A total of 60 clips were selected from the database. These videos included different road types and hazard situations. The clips were edited (using Adobe After Effects CC) to cut to a black screen just as the hazard was beginning to develop. See in S1–S3 Tables, a brief description of the videos used. The clips lasted between 6 and 46 seconds. The clips were displayed on a Toshiba (Satellite Pro) laptop running E-Prime 2.0 Software [44], with a resolution of 1366 x 768 and screen size of 34.5 cm x 19.3 cm.

The walking and riding a bicycle clips were recorded using a Black GoPro Hero5 camera in conjunction with a GoPro Karma-Stabilizer that allows amazingly smooth, shake-free videos. The driving clips were recorded using 4 Silver GoPro Hero4 cameras in order to register the front view and the 3 views available to the driver through the side and rear mirrors. The images from the 4 cameras were merged into one central image with the 3 wing and rear mirrors, simulating real driving. There were no accidents during the recording. There were no accidents during the recording. As supplementary material, a more detailed description of the traffic situations used for the walking, riding a bicycle and driving perspective are shown in S1–S3 Tables.

### Procedure

The test was individually administered in separate cubicles for each participant. After receiving the study information, participants signed the consent form agreeing to take part in the experiment. They were sitting approximately 60 cm from the screen. Some demographic questions were required: age, gender, type of road user, driving experience (year of obtaining the driving license), frequency of driving (miles driven in the past 12 months), and driving collisions in the past 24 months if they considered themselves as vehicle drivers.

After this, the instructions about how to proceed during the experiment were given. Participants carried out 3 sample practice trials. When the task was understood, the experimental trials began. In addition, participants were told to imagine themselves as actors of the clips. The participants were also told that the clips would be cut to black just at the moment when the hazard was starting to develop. A developing hazard can be anything from a pedestrian stepping out into the road, a child running between parked cars, or a car exiting a driveway. It requires to perform an avoiding manoeuvre to prevent the collision. For instance, braking, or change in the speed or direction.

In the first (evaluation) and second block (evaluation and training), 30 different videos from there traffic perspectives were presented: 10 walking, 10 riding a bicycle and 10 driving. The 3 series of 10 clips were presented in random order. The series of 30 videos each one was also counterbalanced between the two blocks. Therefore, a total of 60 videos were presented.

Participants then had to answer 3 questions: Q1. “Did you see the hazard?” No/Yes. Q2. “What might happen next?” Multiple Choice (3 alternatives were proposed to measure SA, only one being what really happened), Q3. “Hazardousness Ratings From 1 to 7” (1 not hazardous at all, 7 very hazardous). After the first block, participants had a 10-minute break. The experiment took an average of approximately 30 minutes.

Accuracy for each participant was calculated from the first two questions. If they answered “no” to the first question (Q1: Did you see the hazard?), their score was 0, and they carried on to the next trial. If the answer was “yes” (I have seen the hazard), then they answered the two following questions: Q2, “What happens next?” (Multiple choice: 1 point for the right choice, 0 points for choosing one of the two distractors). Finally, using a Likert Scale for the anwers, Q3: The Subjective Risk estimation average score was also measured. The Clips Hazardousness Ratings varied from 1 to 7 (1 not hazardous at all, 7 very hazardous). Participants were told that there were no right or wrong answers to this question.

In the second block, 30 new videos were shown. Again 10 new clips from each perspective were shown (10 walking, 10 riding a bicycle, 10 driving) and evaluated. Apart from the assessment task, it was set a combined form of training: Participants were provided with feedback of their execution and with the video outcome (that is., after each trial execution, participants were given “feedback” on their performance, percentage of hits achieved/per video clip visualised; and secondly, the video outcome was provided: a replay of the same video evaluated was displayed, in this second visualisation time, the video was shown again but it was cut a few seconds later, so that the outcome was seen).

## Results

### Internal consistency

This holistic version of the HP and Prediction test showed “good” psychometric reliability. Cronbach’s Alpha coefficient was found to be .846, calculated statistically based on the variance of each question. We also calculated the internal consistency of each of the three subtests and their reliability was found to be acceptable for the walking clips (*α* = .661), riding a bicycle clips (*α* = .658) and driving clips (*α* = .650). Alpha values are sensitive to the number of items in the scale, a sample with a narrow number of items can deflate it. As a rule of thumb, composite reliability values of 0.60 to 0. 70 are acceptable (See Table 2).

**Table 2:** Internal consistency values & cronbach’s alpha. Internal Consistency and Cronbach’s alpha for the video-items used in the HP and Prediction test from the three perspectives of road users.

Walking	Mean	*SD*	Riding a bicycle	Mean	*SD*	Driving	Mean	*SD*
**1**	0.92	*0*.*27*	**21**	0.90	*0*.*30*	**41**	0.90	*0*.*30*
**2**	0.51	*0*.*50*	**22**	0.68	*0*.*47*	**42**	0.57	*0*.*50*
**3**	0.57	*0*.*50*	**23**	0.51	*0*.*50*	**43**	0.70	*0*.*46*
**4**	0.58	*0*.*50*	**24**	0.42	*0*.*50*	**44**	0.94	*0*.*25*
**5**	0.49	*0*.*50*	**25**	0.77	*0*.*42*	**45**	0.70	*0*.*46*
**6**	0.63	*0*.*49*	**26**	0.38	*0*.*49*	**46**	0.75	*0*.*44*
**7**	0.24	*0*.*43*	**27**	0.68	*0*.*47*	**47**	0.67	*0*.*47*
**8**	0.97	*0*.*16*	**28**	0.96	*0*.*19*	**48**	0.73	*0*.*44*
**9**	0.53	*0*.*50*	**29**	0.59	*0*.*49*	**49**	0.76	*0*.*43*
**10**	0.77	*0*.*42*	**30**	0.77	*0*.*42*	**50**	0.87	*0*.*33*
**11**	0.68	*0*.*47*	**31**	0.87	*0*.*33*	**51**	0.70	*0*.*46*
**12**	0.77	*0*.*42*	**32**	0.49	*0*.*50*	**52**	0.92	*0*.*27*
**13**	0.57	*0*.*50*	**33**	0.71	*0*.*46*	**53**	0.57	*0*.*50*
**14**	0.63	*0*.*49*	**34**	0.75	*0*.*44*	**54**	0.66	*0*.*48*
**15**	0.49	*0*.*50*	**35**	0.95	*0*.*22*	**55**	0.53	*0*.*50*
**16**	0.39	*0*.*49*	**36**	0.52	*0*.*50*	**56**	0.84	*0*.*37*
**17**	0.72	*0*.*45*	**37**	0.86	*0*.*35*	**57**	0.77	*0*.*42*
**18**	0.62	*0*.*49*	**38**	0.67	*0*.*47*	**58**	0.92	*0*.*27*
**19**	0.57	*0*.*50*	**39**	0.89	*0*.*32*	**59**	0.89	*0*.*32*
**20**	0.97	*0*.*18*	**40**	0.72	*0*.*45*	**60**	0.80	*0*.*40*
**Total Cronbach’s Alpha: *α* = .846**								

### Q1: Hazard detection (Hits)

A (3)x(2)x4 repeated-measures ANOVA was conducted to examine the differences in Hazard Detection (Hits) between traffic perspectives (walking, riding a bicycle and driving) and block types (B1, no training and B2, training) as the two within-subjects factors, and the different road user profiles (pedestrian, cyclist, novice and experienced driver) as the between-subjects factor.

**Fig 2:**
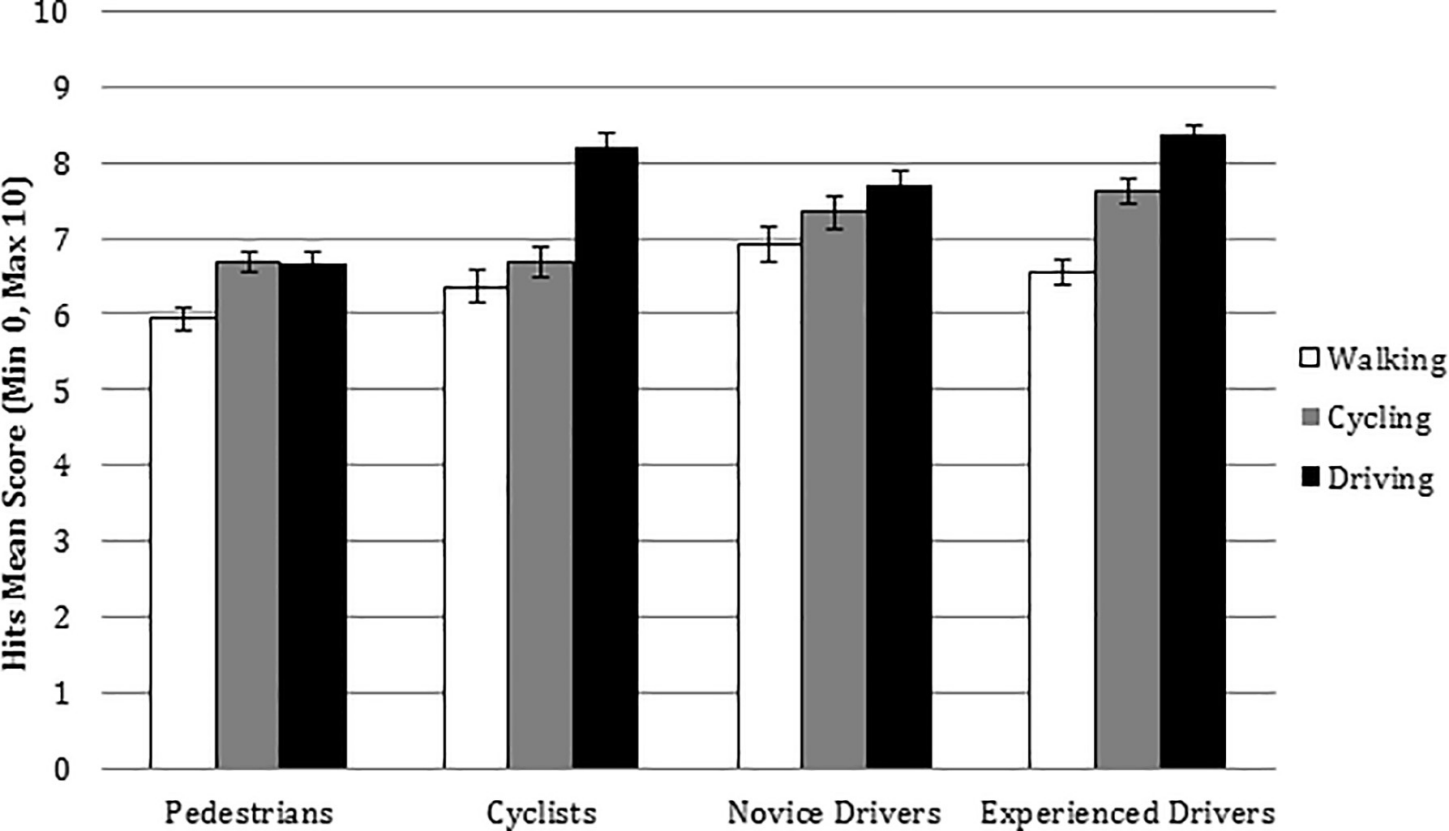
Interaction road users X traffic perspectives: HP and Prediction scores. HP and Prediction Hits mean score for road users (pedestrian, cyclist, novice and experienced driver) and traffic perspectives (walking, riding a bicycle and driving). Error bars represent standard errors.

Two interactions were found to be significant: the interaction between traffic perspective and road user [*F*(6,150) = 3.461, *p* = .003, *η*^2^_p_ = .122, BF_10_ = 4.644], and traffic perspective and type of block [*F*(2,150) = 5.030, *p* = 0.008, *η*^2^_p_ = .063, BF_10_ = 10.299], so we proceeded to analyse these interactions. When analysing the pairwise comparisons, these differences were revealed to be statistically significant differences in the driving perspective score between pedestrians (*M* = 6.683, *SE* = .241) and cyclists (*M* = 8.214, *SE* = .352); and pedestrians and experienced drivers (*M* = 8.364, *SE* = .281) (see Fig 2).

The three main effects were found to be significant: A significant main effect of traffic perspective [*F*(2,150) = 34.968, *p* < .001, *η*^2^_p_ = .318, BF_10_ = 5.599e+10] (walking M = 6.44, SE = 1.75, riding a bicycle *M* = 7.08, SE = 1.65 and driving clips *M* = 7.47, SE = 1.31). Pairwise comparisons showed statistically significant differences between the traffic perspectives walking (*M* = 6.44) and riding a bicycle (*M* = 7.08) (*p* < .001) and also between walking (*M* = 6.44) and driving clips (*M* = 7.47) (*p* < .001)

A significant main effect of road user [*F*(3,75) = 3.335, *p* = .024, *η*^2^_p_ = .118, BF_10_ = 3.911]: (pedestrians, M = 5.82, SE = .21, cyclists, M = 6.39, SE = 1.23, novice (M = 6.89, SE = .85), and experienced drivers (M = 7.07 SE = 1.13).

And a significant main effect of block type [*F*(1,75) = 146.400, *p* < .001, *η*^2^_p_ = .661, BF_10_ = ∞] (B1, no training, M = 5.82, SE = .21 vs. B2, training, M = 6.39, SE = 1.23)

**Fig 3:**
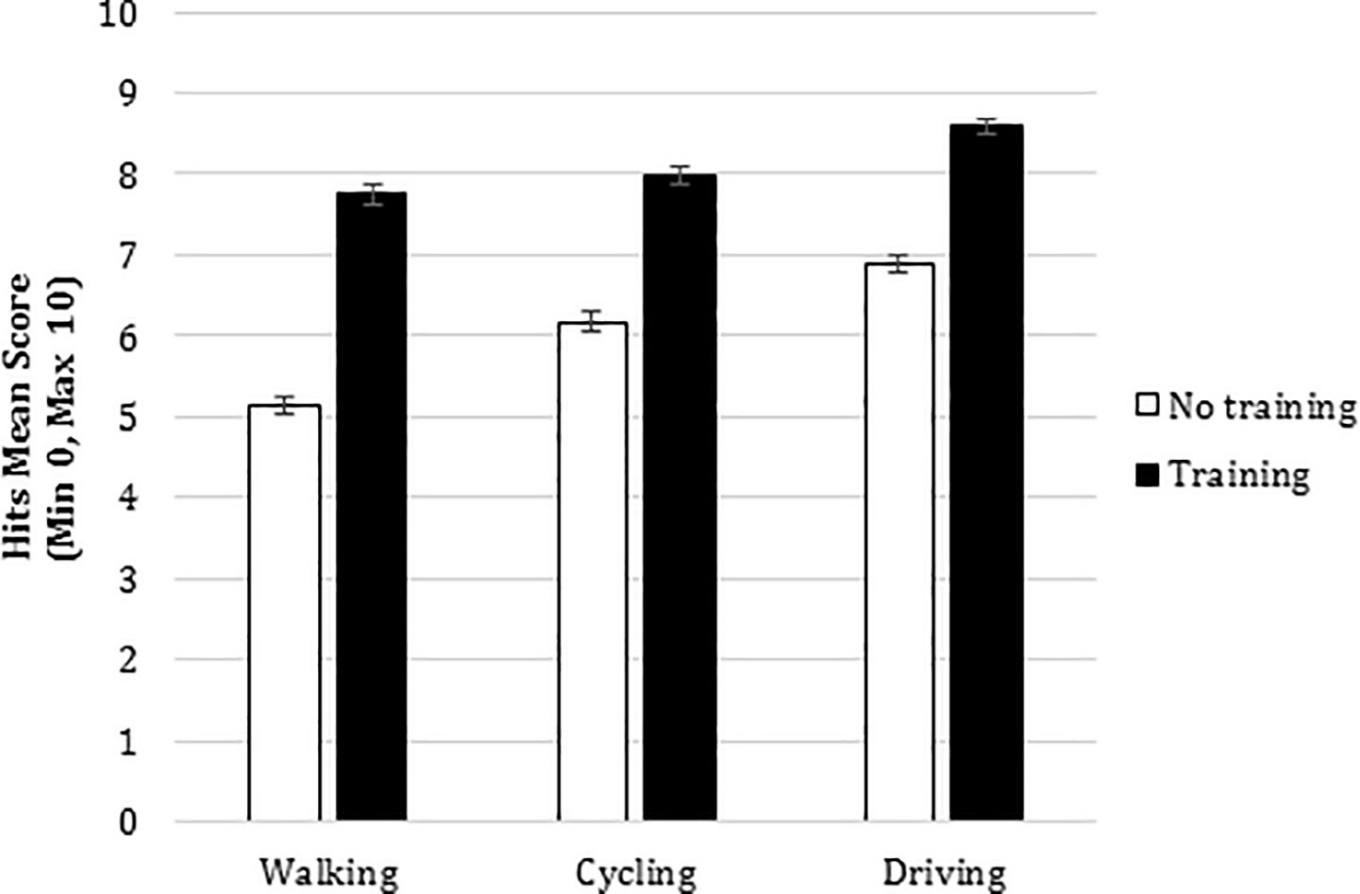
Interaction type of block X traffic perspectives: HP and Prediction scores. HP and Prediction Hits mean scores for type of block (No training and Training block) and traffic perspectives (walking, riding a bicycle and driving). Error bars represent standard errors.

As for the second interaction, paired comparisons revealed significant differences on walking clips between the no training (*M* = 5.133, *SE* = .227) and the training blocks (*M* = 7.746, *SE* = .23), in the riding a bicycle clips (untrained *M* = 6.173, *SE* = .234; trained *M* = 7.998, *SE* = .206) and in the driving clips (untrained *M* = 6.884, *SE* = .211; trained *M* = 8.592, *SE* = .19). (See Fig 3)

### Q2: HP and prediction (Situation Awareness)

A (3) x (2) x 4 repeated-measures ANOVA was conducted to examine the differences in HP and Prediction (SA) between traffic perspectives (walking, riding a bicycle and driving) and block type (B1, no training and B2, training) as within-subjects factors, and the different road user profiles (Pedestrian, Cyclist, Novice driver and Experienced driver) as the between-subjects factor (see Table 3).

**Table 3:** Situation Awareness scores. HP and Prediction accuracy (Situation Awareness) in the experimental conditions manipulated for the Hits answered as ‘Yes, I did see the hazard’.

SITUATION AWARENESS		No training (B1)	Training (B2)		
Road User	Clips	Mean *(SD)*	Mean *(SD)*		
	Walking	**1.20**	**2.53**	**1.87**	
		*(0*.*89)*	*(1*.*38*	*(1*.*14)*	
Pedestrians	Riding a Bicycle	**1.50**	**2.67**	**2.09**	**2.06**
		*(1*.*20)*	*(1*.*24)*	*(1*.*22)*	*(1*.*24)*
	Driving	**2.07**	**2.37**	**2.22**	
		*(1*.*26)*	*(1*.*47)*	*(*1.37*)*	
	Walking	**1.86**	**2.71**	**2.29**	
		*(1*.*10)*	*(0*.*99)*	*(*1.05*)*	
Cyclist	Riding a Bicycle	**2.07**	**2.71**	**2.39**	**2.46**
		*(1*.*00)*	*(1*.*14)*	*(1*.*07)*	*(1*.*14)*
	Driving	**2.50**	**2.93**	**2.72**	
		*(1*.*51)*	*(1*.*07)*	*(1*.*29)*	
	Walking	**1.92**	**3.46**	**2.69**	
		*(1*.*44)*	*(1*.*39)*	*(*1.42*)*	
Novice Drivers	Riding a Bicycle	**2.15**	**3.15**	**2.65**	**2.64**
		*(1*.*95)*	*(1*.*72)*	*(1*.*84)*	*(1*.*48)*
	Driving	**2.00**	**3.15**	**2.58**	
		*(1*.*22)*	*(1*.*14)*	*(1*.*18)*	
	Walking	**1.68**	**2.68**	**2.18**	
		*(1*.*29)*	*(1*.*17)*	*(*1.23*)*	
Experienced Drivers	Riding a Bicycle	**2.41**	**2.73**	**2.57**	**2.51**
		*(0*.*91)*	*(1*.*39)*	*(1*.*15)*	*(1*.*28)*
	Driving	**2.59**	**2.95**	**2.77**	
		*(1*.*40)*	*(1*.*53)*	*(1*.*47)*	
		**2.00**	**2.84**		
		*(1*.*26)*	(1.30)		

In relation to SA, significant main effects of traffic users [*F*(3,75) = 4.079, *p* = .010, *η*^2^_p_ = .140, BF_10_ = .536] and block type [*F*(1,75) = 46.339, *p* < .001, *η*^2^_p_ = .382, BF_10_ = 2.358e+9] were found. Significant interactions were not found.

For road users, Bonferroni’s test post hoc multiple comparisons analysis revealed significant differences between pedestrians (*M* = 2.056, *SE* = .11) and novice drivers (*M* = 2.641, *SE* = .167).

For blocks, Bonferroni’s test post hoc multiple comparisons analysis revealed significant differences between untrained (*M* = 1.996, *SE* = .095) and trained blocks (*M* = 2.838, *SE* = .095)

#### Subjective risk estimation

A (3) x 4 repeated-measures ANOVA was conducted to examine the differences in Risk Estimation between traffic perspectives (walking, riding a bicycle and driving) as the within-subjects factor, and the different traffic user profiles (Pedestrian, Cyclist, Novice Driver and Experienced driver) as the between-subjects factor. Risk estimation score was calculated from estimates in the videos where participants had previously answered “yes” to the first question. No violations of assumed sphericity were found for this analysis.

**Fig 4:**
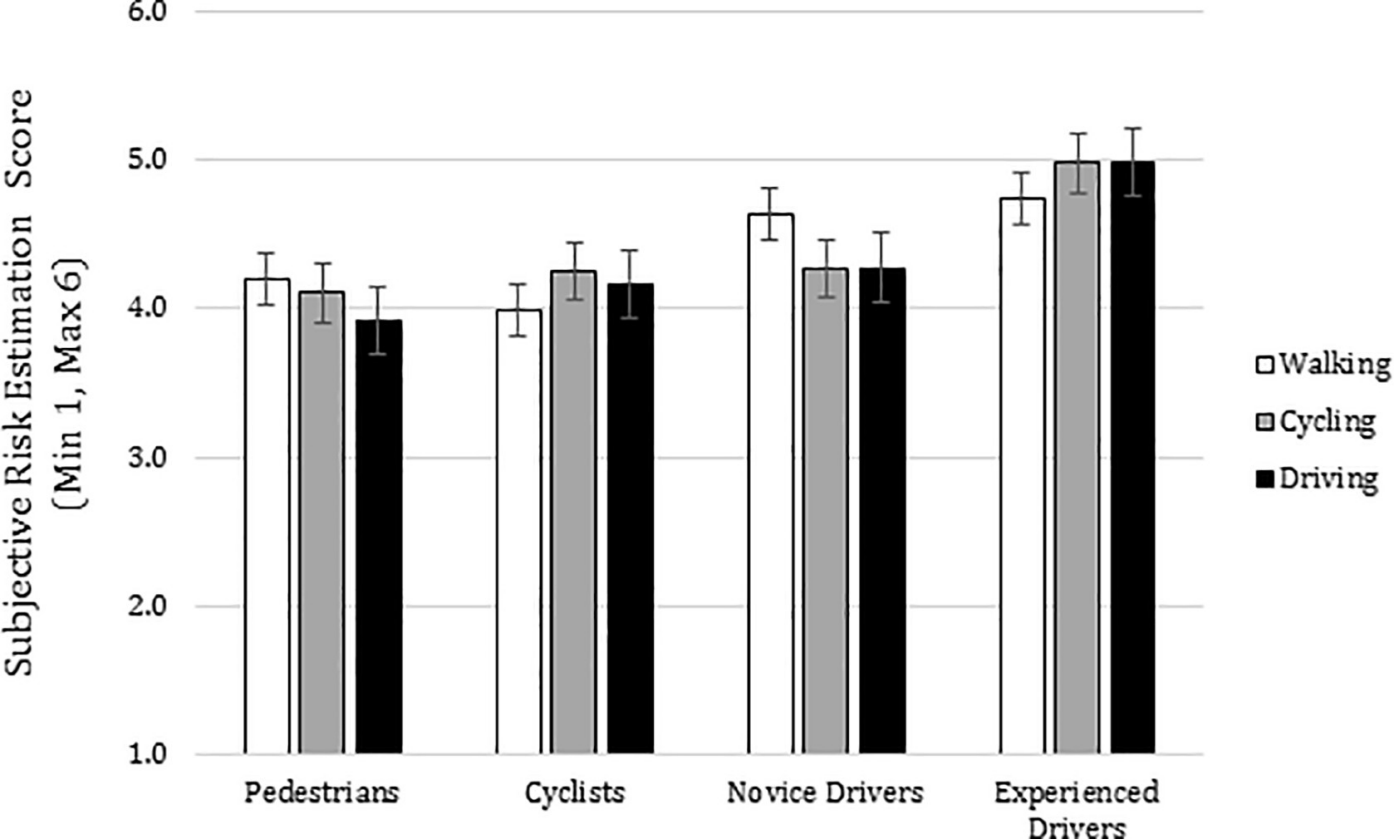
Interaction road users X traffic perspectives: Risk estimation scores. Subjective risk estimation mean scores for road users (pedestrians, cyclists, novice and experienced drivers and for traffic perspectives (walking, riding a bicycle and driving). Error bars represent standard errors.

With regard to risk estimation, we found a significant interaction between traffic perspective and road user [Sphericity assumed *F*(6,150) = 3.709, *p* = .002, *η*^2^_p_ = .129, BF_10_ = .417]. In addition, we found a significant main difference between road users [*F*(3,75) = 3.876, *p* = .012, *η*^2^_p_ = .134, BF_10_ = 10.302] (See Fig 4).

Pairwise comparisons across walking clips revealed significant differences between cyclists (*M* = 3.99, *SD* = .796) and experienced drivers (*M* = 4.736, *SD* = 1.197). Pairwise comparisons across riding a bicycle clips revealed significant differences between pedestrians (*M* = 4.107, *SD* = .905) and experienced drivers (*M* = 4.981, *SD* = 1.044) and between cyclists (*M* = 4.253, *SD* = .775) and experienced drivers. Pairwise comparisons across driving clips revealed significant differences between pedestrians (*M* = 3.922, *SD* = .922) and experienced drivers (*M* = 4.987, *SD* = 1.159)

## Discussion

We have succeeded in creating a new holistic test that measures the skills of HP and Prediction and risk estimation, and has good psychometric properties that discriminate 1) between various traffic hazards recorded from different perspectives: walking, cycling and driving a car, 2) between participants with different road user profiles (pedestrians, cyclists and drivers) and 3) between two test blocks, the first evaluation only and the second combining evaluation and intervention.

Specifically, the participants had a worse performance, Hits and SA (Perception, Comprehension and Prediction) in the videos recorded walking than in those recorded cycling, while the best scores were found in the videos recorded when driving a car. As it was said before, many make the assumption that the “not having seen” a pedestrian means that the pedestrian or bike was difficult to see [39]. However, it could also be because the attention of vehicle drivers was focused on other drivers. In turn, pedestrians showed the worst results in detection and in SA of all the hazards. Pedestrians fail to attend to potential dangers involving the behaviour of other road users, in contrast to experienced drivers, who increase their visual scanning on roadways of increased complexity. Modal bias affects HP on the road. Up-down processes influence our visual search. Expectations and previous experience guide our attention. We see what we want to see [45]. The number of events to which we can give our attention is limited. The number of events to which we can give our attention is limited. We are bombarded by more information than we are able to process and it is attention that is the key to accessing our awareness, filtering the information we think is most interesting.

We also found that the training in change of perspective given in the second block was useful in enabling all road users to improve their detection and SA of the hazards recorded from different perspectives (walking, cycling or driving) and this improvement would be particularly large in the case of hazards recorded walking. Thus, the usefulness of the analysis of change of perspective is demonstrated, not only as a mode of evaluation but also as a form of training in the skill of HP for different road users. The advantage found in the results of the second block could be due either to the participants carrying out the evaluation task with a greater number of videos (effect of practice) or to them receiving feedback on their performance and the outcome of the video.

Regarding Risk Estimation, again an effect of modal bias was found: Drivers underestimated the risk in walking situations more than in the situations driving a car. According to our results, it is also true that pedestrians underestimated the danger driving a car more than in walking situations.

In summary, to mitigate the difficulties in detection of vulnerable users [39], it is necessary to improve Detection and SA of traffic hazards when we are walking, cycling or driving a vehicle and avoid distraction [46]. If this improvement were achieved, we might become more conscious of all road users, including the most vulnerable (pedestrians, cyclists), and equally, they would improve their HP skill.

Specifically, holistic training that manages to put road users in the place of other users (actor-observer) enriches SA and improves the skill of HP and Prediction (i.e. identifying, recognising and reacting to potentially dangerous traffic situations), which correlates with the avoidance of accidents [1]. It may also have other collateral positive effects: A) serving to reduce the psychological egoism with which we drive [47] mitigating the effect of the “insecurity of low numbers as a probabilistic effect that is detrimental to users of transport less likely to be circulating on the road [40, 41], C) reducing the social modal biases that, when applied to traffic, also distort the interpretation of situations and influence decision-making [47].

### General conclusions

The use of different perspectives in one Holistic HP and Prediction test might increase the generalizability and wider use of method in future research and practice. A more empathic vision of traffic could be useful to improve Detection and Prediction of hazards, which might mitigate, at least partly, the modal biases that contribute to accidents, above all because the dangers from some traffic perspectives (such as walking) are more difficult to predict and because of the difficulty some users (such as pedestrians) generally have in detecting traffic dangers. Holistic training would also be positive in order to avoid modal biases that cause underestimation of risk from traffic perspectives other than those normally preferred by the road user. This raises the possibility of planning distinct forms of training that might generate new strategies of visual processing that could help participants put themselves in the place of other road users. It is possible to create memory tracks and to form strong expectations that would enable road users to identify future traffic hazards more quickly and precisely, in both recorded situations and in real driving. In addition, the aim of a new study could be to differentiate the effects of practice per se from different types of training.

## Supporting information

S1 Table Walking clips. A description of hazards selected for walking traffic perspective’ clips.
(DOCX)

S2 Table Cycling clips. A description of hazards selected for cyclist traffic perspective’ clips.
(DOCX)

S3 Table Driving clips. A description of the hazards selected within vehicle traffic perspective’ clips.
(DOCX)
