# Supplementary material for: Hazard Perception and Prediction test for walking, riding a bike and driving a car: “Understanding of the global traffic situation”
Source: PLoS One. 2020 Oct 16;15(10):e0238605. doi: 10.1371/journal.pone.0238605 (PMC7567349; doi:10.1371/journal.pone.0238605)
Supplement: S1 Table — A description of hazards selected for walking traffic perspective’ clips. (DOCX) [file pone.0238605.s001.docx]

**S1 Table. Walking Clips**

A description of hazards selected for walking traffic perspective’ clips

| Nº/Fig. | Walking clips | (sec.) | Last sketch,  previous to the clip occlusion | Hazard |
| --- | --- | --- | --- | --- |
| Walking_1 | You are walking along the sidewalk, coming to the zebra crossing to cross the street. When you are arriving, many vehicles are going through the zebra crossing. The clip occludes when you step on the road | 14’ | 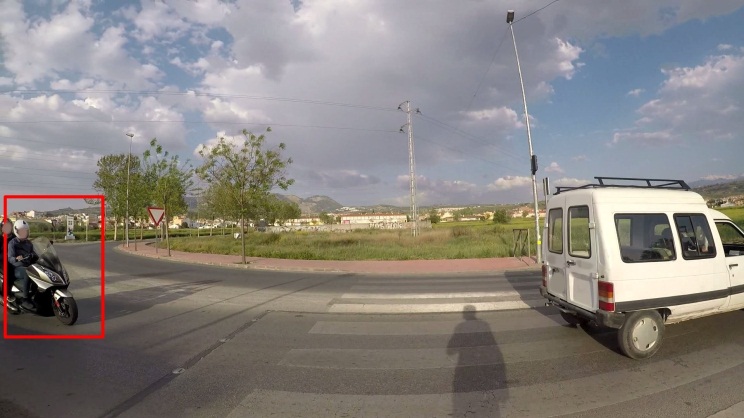 | A motorcycle crosses the zebra crossing from the left |
| Walking_2 | You approach to the zebra crossing, with the intention to cross it. The clip occludes at the point of one car is arriving to the zebra crossing from the left, and you are about to cross it | 10’ | 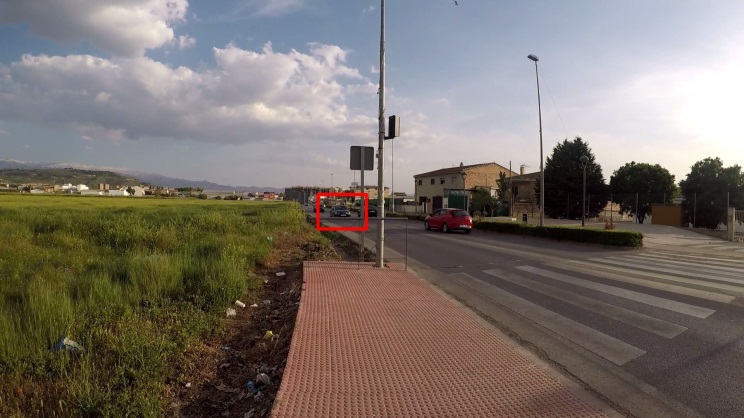 | A car is arriving from the left to the zebra crossing |
| Walking_3 | Walking along the city, you turn right and go towards the pedestrian crossing. The clip occludes just when traffic lights change from red to green | 16’ | 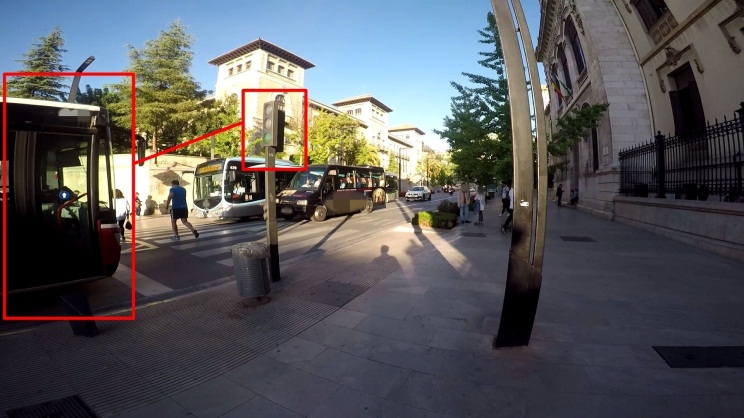 | The traffic light turn to green and vehicles begin to move |
| Walking_4 | You begin to cross the street when suddenly you observe that a white car is coming to you. The clip occludes when the car is a few meters of distance to the pedestrian crossing | 15’ | 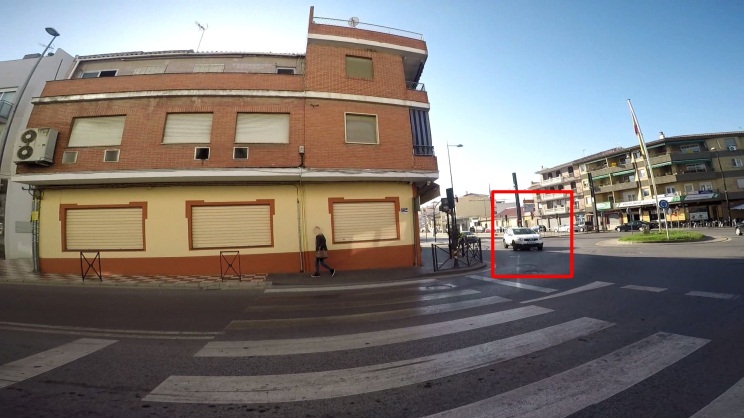 | A car from the right is going to cross the zebra crossing |
| Walking_5 | As you are walking in direction to the zebra crossing, a car is approximating to it also. The clip occludes when the car is coming to the advance stop line | 10’ | 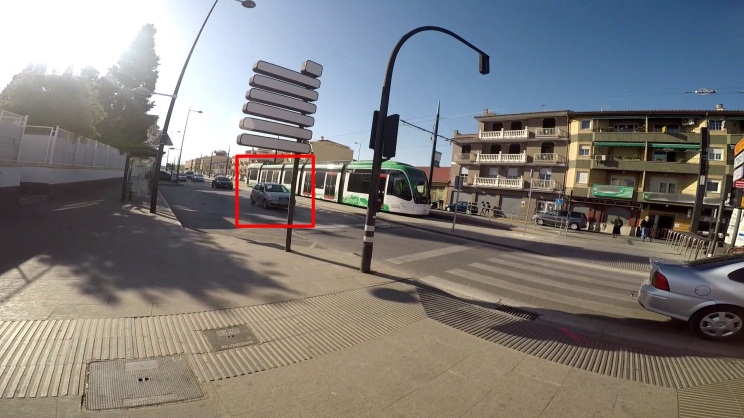 | A car from the left is going to cross the zebra crossing |
| Walking_6 | Trying to cross a congested street, you begin to go through the pedestrian crossing while a car is crossing it from the left to the right. A car behind the first one is crossing the street along the another lane from the right to the left, and the clip occludes when this second car is partially visible | 7’ | 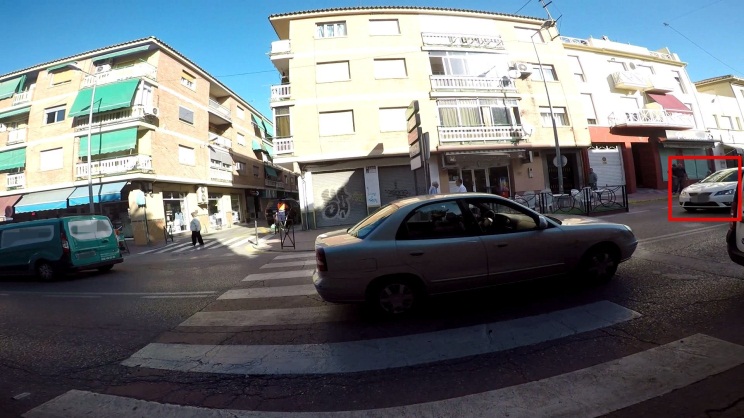 | A half-occluded car from the right is about to cross the zebra crossing |
| Walking_7 | You head for a two-way zebra crossing, divided by a central reservation. Before you arrive to the street, two cars are approaching to the zebra crossing. The clip occludes when one of them is about to cross the path | 13’ | 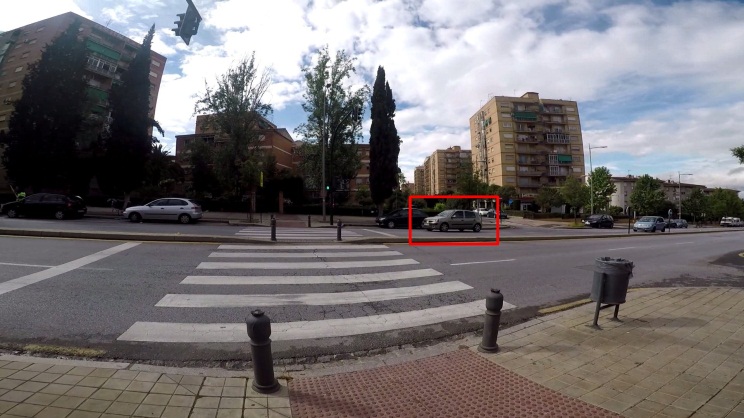 | A car from the right is about to cross the zebra crossing |
| Walking_8 | Walking along the sidewalk, you turn right and go towards the street, with the intention to cross it. There is a parked van to your left that hinder you from seeing the motorcycle that comes from the left. The clip occludes just when the motorcycle is completely visible | 16’ | 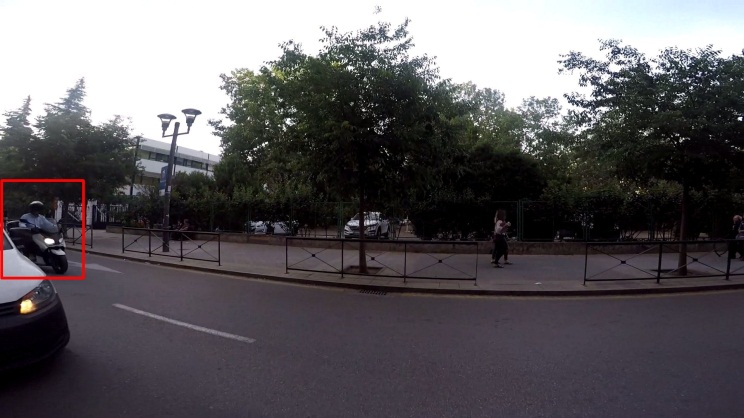 | A motorcycle is traveling along the street you are crossing |
| Walking_9 | You are walking along the pavement and turn right with the intention to cross the street. When you begin to cross the road, a car from the left is going to cross the zebra crossing. The clip occludes when the car comes to the advance stop line | 9’ | 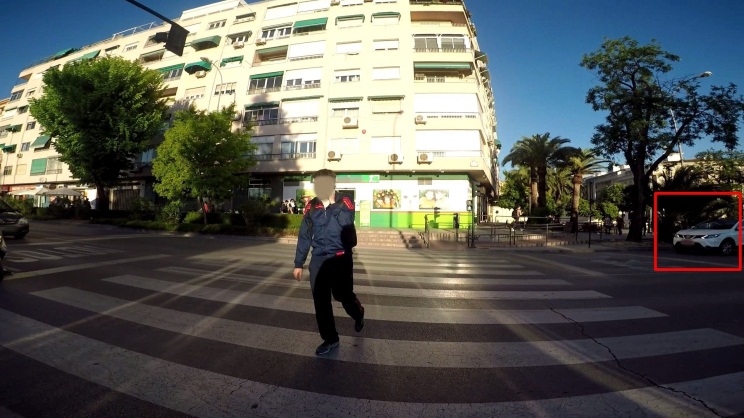 | A car from the right is about to cross the zebra crossing |
| Walking_10 | Strolling in the street, you decide to turn left and cross the street. In that particular moment, a motorcycle shows up from the left, whit the intention to cross the zebra crossing. The clip occludes when the motorcycle turn left to traverse the zebra crossing | 18’ | 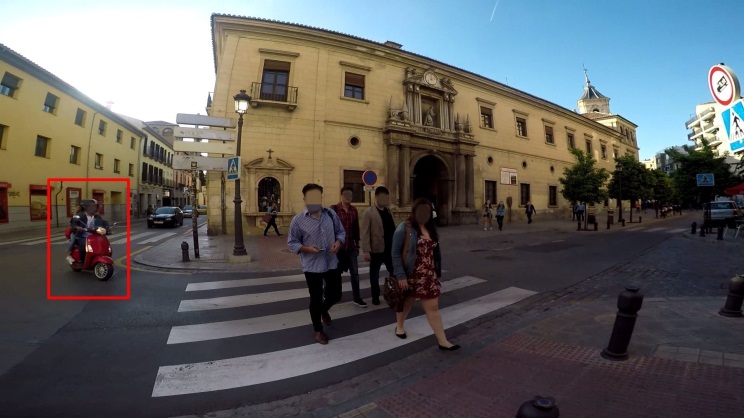 | A motorcycle from the left is about to cross the zebra crossing |
| Walking_11 | Walking along the city, you need to cross the pedestrian crossing to continue with your route. At the moment you are crossing, a grey car turns slightly right to go across the zebra crossing. The clip occludes when the manoeuvre of the car is perceptible | 10’ | 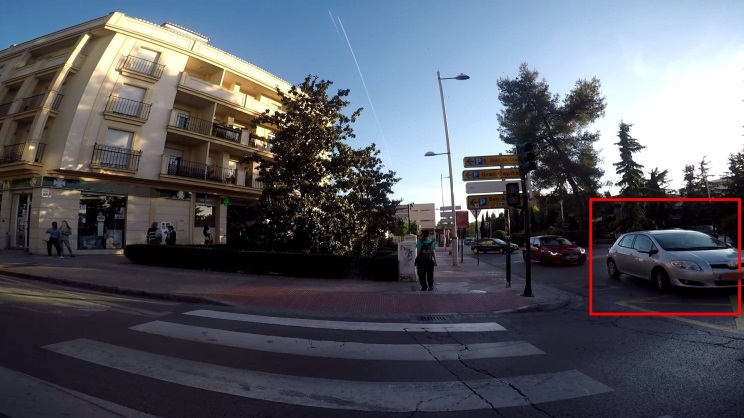 | A car turn in direction to the zebra crossing to cross it |
| Walking_12 | You are walking along the sidewalk when you turn left to cross the street through the zebra crossing. After you step on it, a car shows up from the right. The clip occludes when the headlamps of the car are perceptible | 13’ | 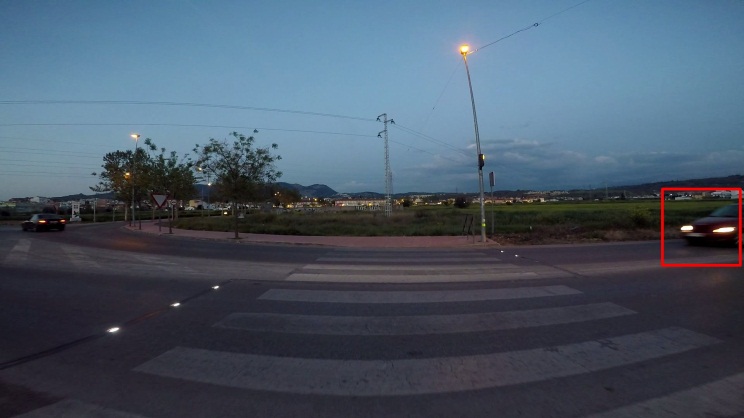 | A car from the right is about to go through the zebra crossing |
| Walking_13 | After you leave a terrace restaurant behind, you turn left with the intention to cross the street. When you are about to do it, a car from the right is approaches dangerously to the zebra crossing | 7’ | 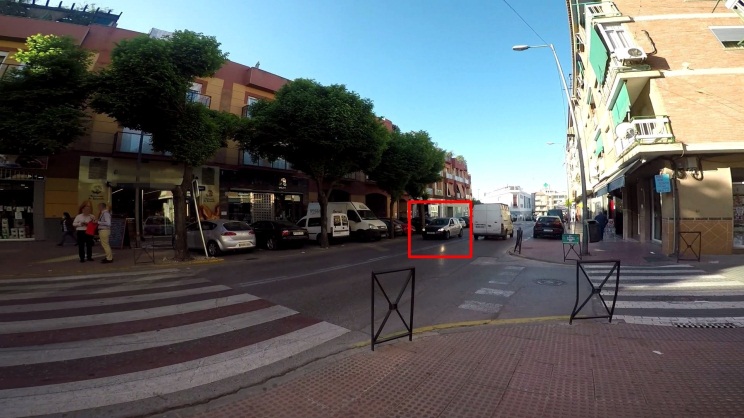 | A car from the right is about to cross the zebra crossing |
| Walking_14 | Walking along the town, you turn right with the intention to cross the street. As you approach to the zebra crossing, a car is arriving to it. The clip occludes when the car is about to cross the advance stop line | 10’ | 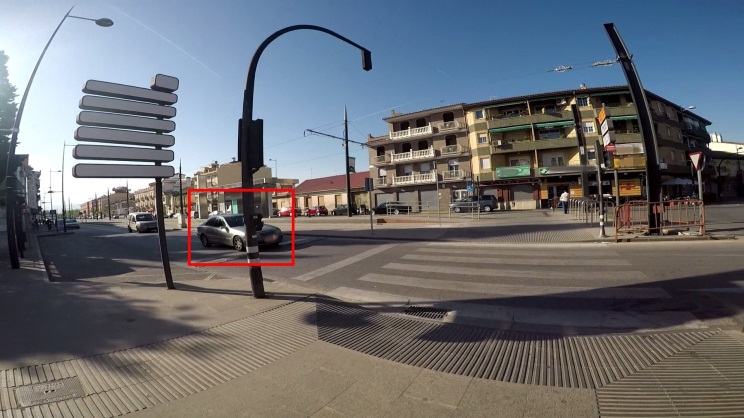 | A car is about to cross the advance stop line from the left |
| Walking_15 | You are scrolling in the town when you turn left to cross the street. At that exact moment, a motorcycle is about to cross the zebra crossing without the intention to stop. The clip occludes just at the point where the motorcycle is about to step on the zebra lines | 11’ | 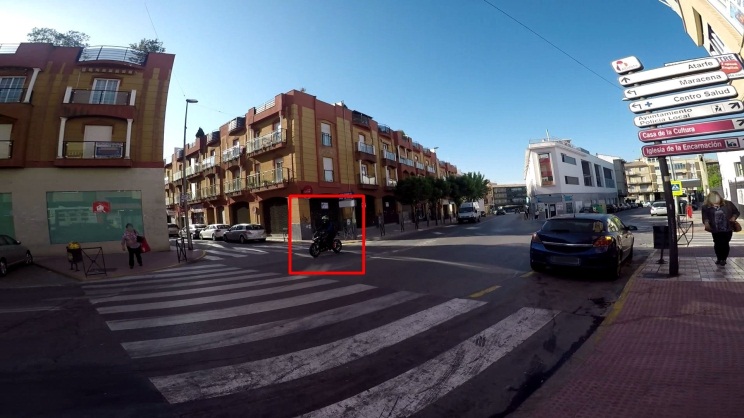 | A motorcycle is approaching to the zebra crossing |
| Walking_16 | Walking along the pavement, you turn left to move to the contrary sidewalk of the street. As you do it, a car approaches from the right, but the trash bins hides it from your vision. The clip occludes when the car show up from behind the trash bins | 9’ | 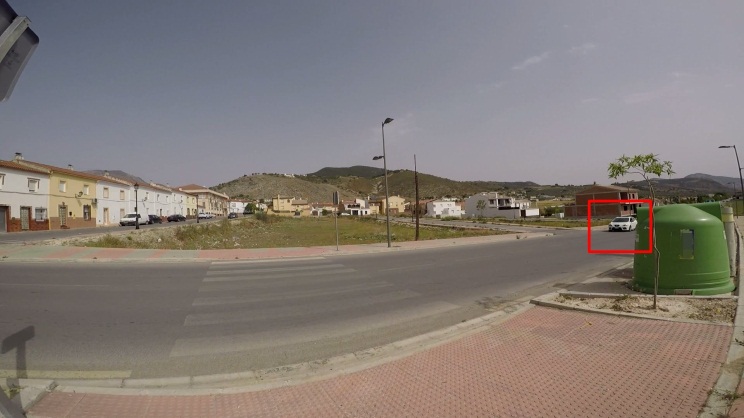 | A car is going to cross the zebra crossing from the right |
| Walking_17 | You are walking along the sidewalk. Then, you turn right to cross the street. When you step on the zebra crossing, a car show up from the right side of the road. The clip occludes when the first half of the car is visible | 12’ | 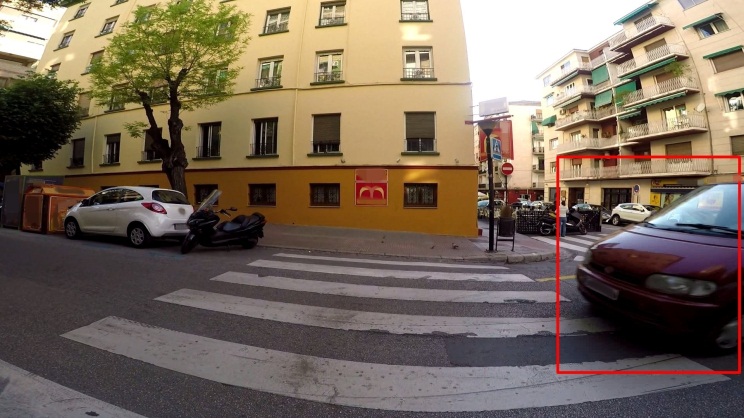 | A car cross the zebra crossing from the right |
| Walking_18 | You are going to cross the street while the traffic light is flashing amber. The clip occludes just when you step on the zebra crossing and a motorcycle show up with the intention to cross too | 8’ | 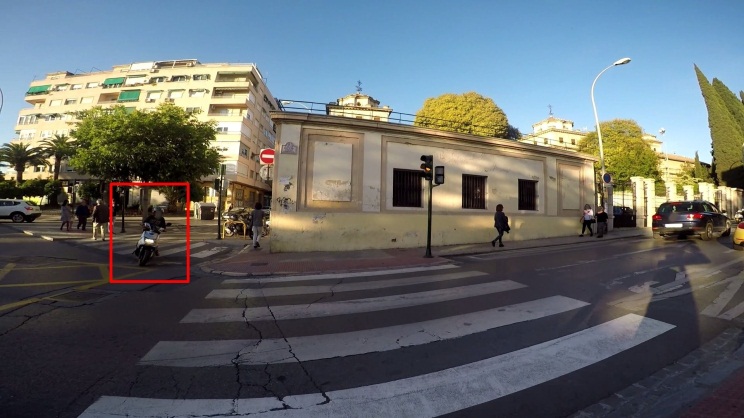 | A motorcycle is about to cross the zebra crossing while the traffic light is flashing amber |
| Walking_19 | You are walking along a narrow pavement. After several steps, you turn left to go towards the hospital entrance, for which it is necessary to cross the street. When you begin to cross the zebra crossing, a car approaches to you from the right. The clip occludes just in that moment | 19’ | 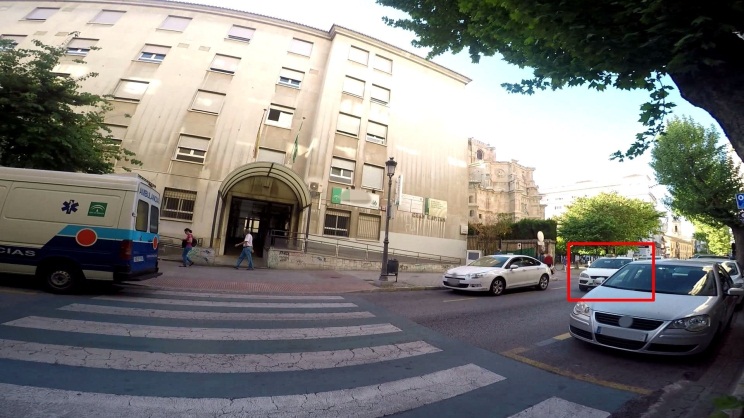 | A car is approaches to the zebra crossing from the right |
| Walking_20 | First of all, you turn left and cross a zebra crossing. Then, you turn right and approach to the next zebra crossing. When you are close to it, a pedestrian occlude the vision of the zebra crossing partially. The clip occludes just when a van cross the path from the left | 18’ | 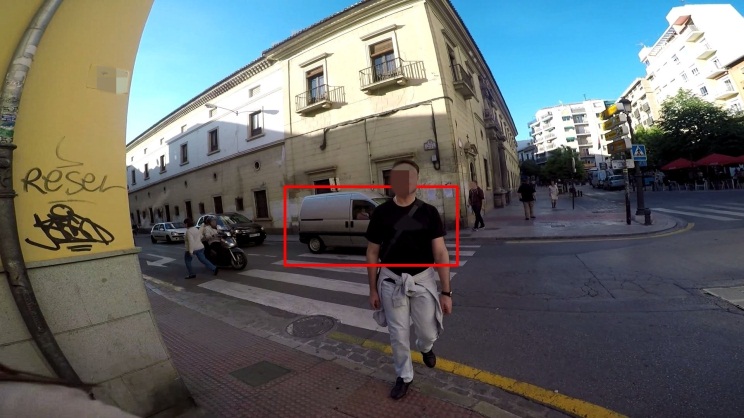 | A van cross the zebra crossing from the left just when you are going to begin to cross it |
