# Supplementary material for: Hazard Perception and Prediction test for walking, riding a bike and driving a car: “Understanding of the global traffic situation”
Source: PLoS One. 2020 Oct 16;15(10):e0238605. doi: 10.1371/journal.pone.0238605 (PMC7567349; doi:10.1371/journal.pone.0238605)
Supplement: S2 Table — A description of hazards selected for cyclist traffic perspective’ clips. (DOCX) [file pone.0238605.s002.docx]

**S2 Table. Cycling Clips**

A description of hazards selected for cyclist traffic perspective’ clips

| \| Nº/Fig. \| Cycling clips \| (sec.) \| Last sketch,  prior the clip occlusion \| Hazard \| \| --- \| --- \| --- \| --- \| --- \| \| Cycling_1 \| You are bicycling along a boulevard road, at the right lane. The clip occludes when a dark car try to change lane from the other one \| 18’ \| 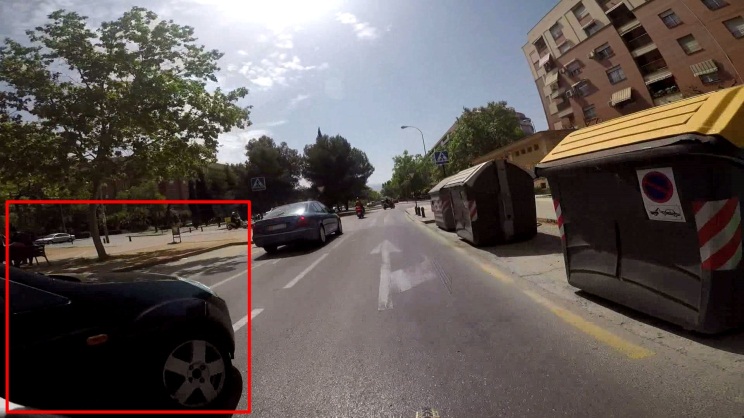 \| A car gets into your lane from the left \| \| Cycling_2 \| Bicycling along a two-way cycle lane, you can see many pedestrians on the sidewalk. The clip occludes when one pedestrian is almost crossing the cycle lane \| 19’ \| 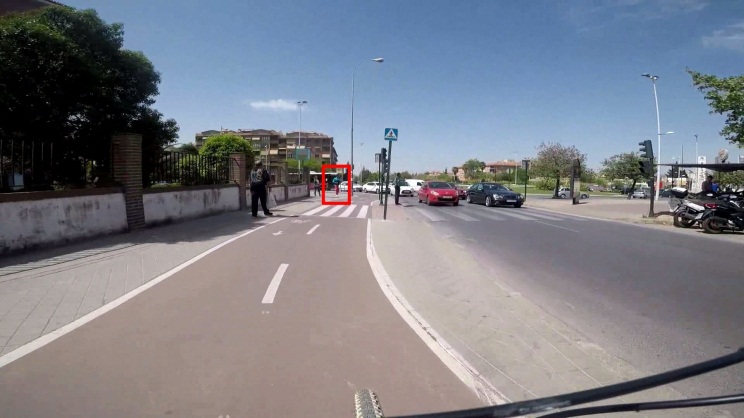 \| A pedestrian crosses the cycle lane \| \| Cycling_3 \| As you are traveling along a cycle lane towards an intersection, a yellow car show up from the right side. The clip occludes the car is completely perceptible \| 15’ \| 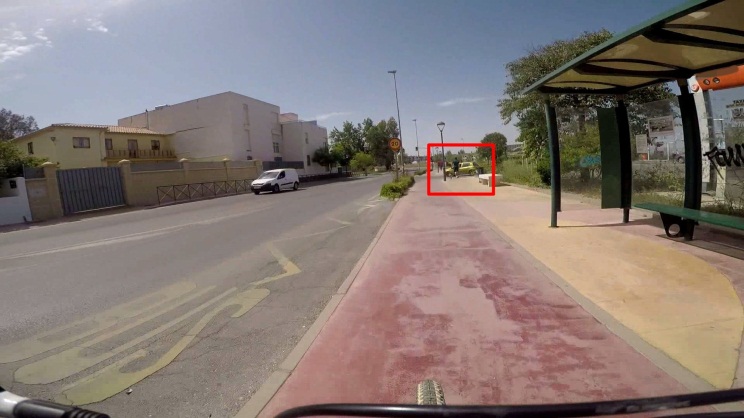 \| A car come across the cycle lane \| \| Cycling_4 \| You turn right towards a two-way urban road. In that moment, the traffic light is on amber but it turn to red, and a pedestrian has the intention to cross the street. The clip occludes just when traffic light turn to red \| 3’ \| 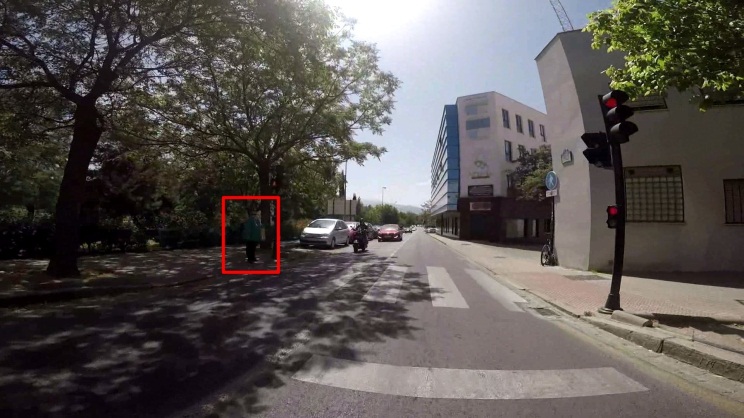 \| The traffic light turn to red and a pedestrian prepares to cross the street \| \| Cycling_5 \| Traveling along a two-way cycle lane, you are coming to a stop signal because of an intersection. A car has the intention to cross the cycle lane from the right lane of the intersection, and a pedestrian is between both you and the car. The clip occludes when the car is hidden behind the pedestrian \| 10’ \| 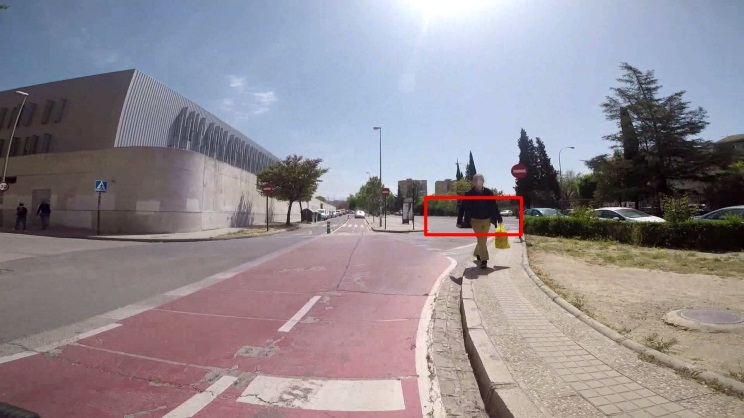 \| A car is about cross the cycle lane \| \| Cycling_6 \| Bicycling along a boulevard road, a bottleneck showed up few meters ahead. The clip occludes when the car at the bottom of the jam is perceptible \| 26’ \| 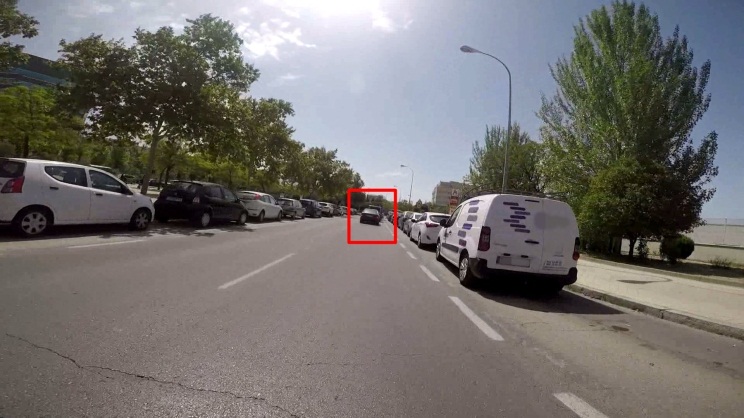 \| A bottleneck on a boulevard road \| \| Cycling_7 \| You are traveling along a town road. After taking the last curve, you come to a zebra crossing. The clip occludes when two pedestrian start to cross the street few meters ahead. \| 17’ \| 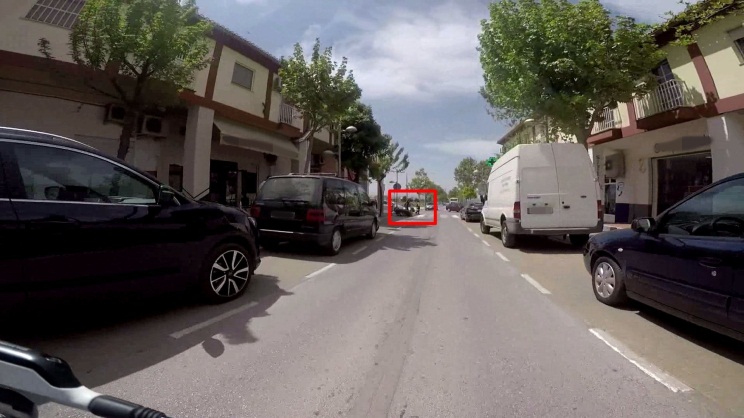 \| Two pedestrians from the right sidewalk cross the street \| \| Cycling_8 \| Bicycling along an urban road, you observe a green car double-parked at the right side. Because it is on your lane, you have the intention to change lane, but in the moment you turn left, a car overtakes you from the lane you go to. The clip occludes just in that moment. \| 41’ \| 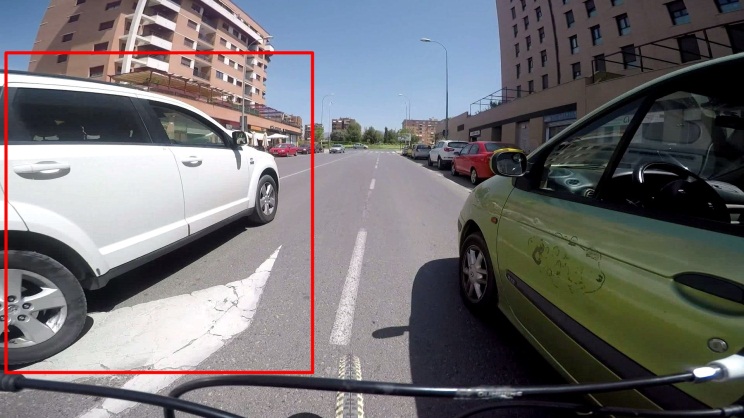 \| A car overtakes you from the lane you are getting into. \| \| Cycling_9 \| Riding a bike on a boulevard road, you travel towards a traffic light. Moments before coming there, a pedestrian show up between the parked cars on the right. The clip occludes as the pedestrian is about to cross the street. \| 19’ \| 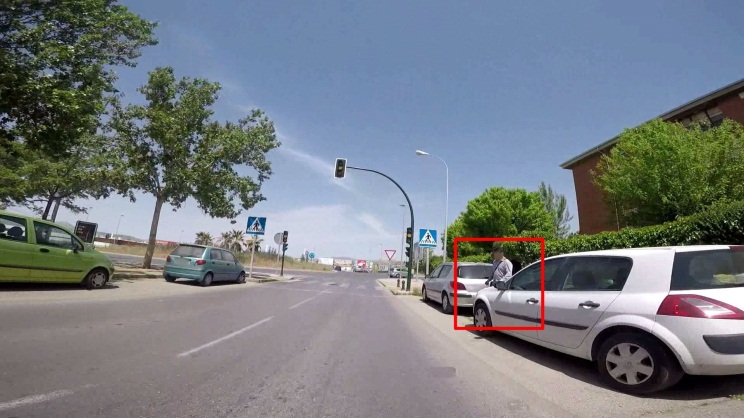 \| A pedestrian from the right is about to cross the street \| \| Cycling_10 \| Traveling uphill on a town road, you observe a white car braking. As you are coming close to it, the car starts to go in reverse. The clip occludes following one flash of the backup lights from the car \| 5’ \| 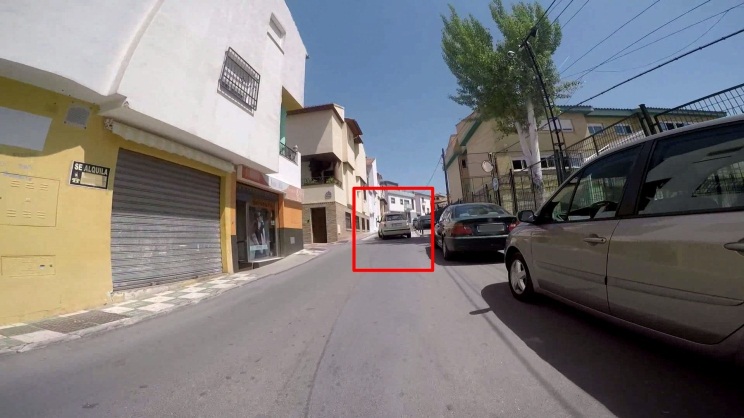 \| A car go in reverse into your lane \| \| Cycling_11 \| You are bicycling along a boulevard road. Coming to a zebra crossing, a pedestrian and her pet show up from behind a white van. The clip occludes just in that moment, when they begin to cross the street \| 32’ \| 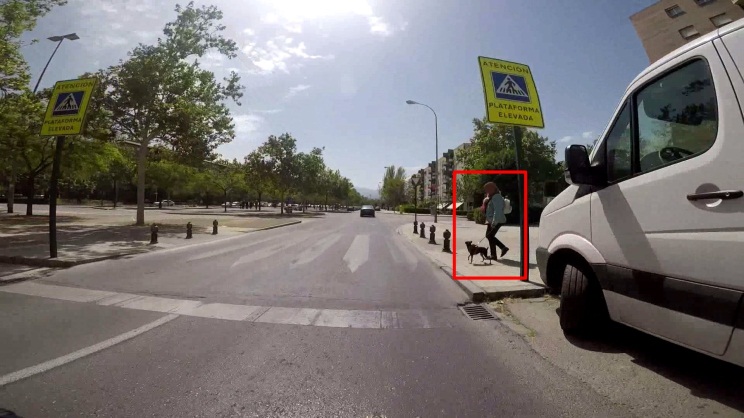 \| A pedestrian and her dog are about cross the street \| \| Cycling_12 \| You ride a bike along a two-way cycle lane. A pedestrian look likes to cross the cycle lane from the left side. The clip occludes just as he steps on the cycle lane \| 14’ \| 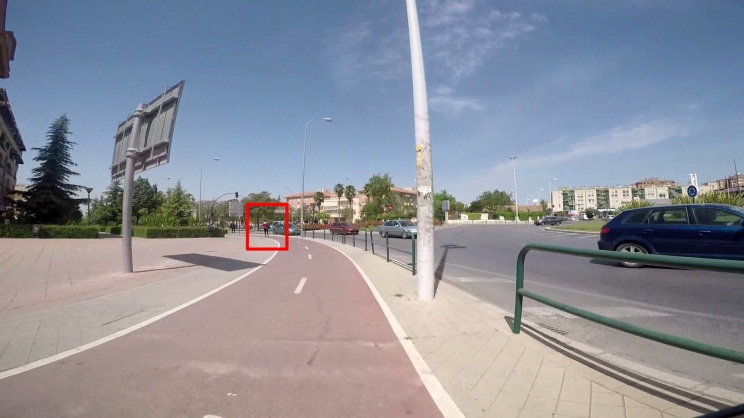 \| A pedestrian from the left crosses the cycle lane \| \| Cycling_13 \| Traveling along a two-way urban road, there is parked cars on both sides. At the moment you cross the pedestrian crossing, a parked car put the bonnet out your lane. The clip occludes when the bonnet is completely visible \| 23’ \| 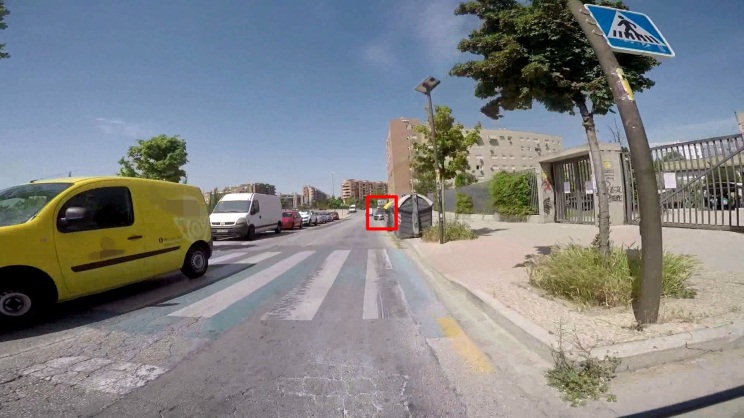 \| A parked car gets into your lane from the street parking \| \| Cycling_14 \| As you are bicycling on a cycle lane, a bus stops a few meters ahead, at the left side. The clip occludes when a passenger gets off the bus and steps on the cycle lane \| 13’ \| 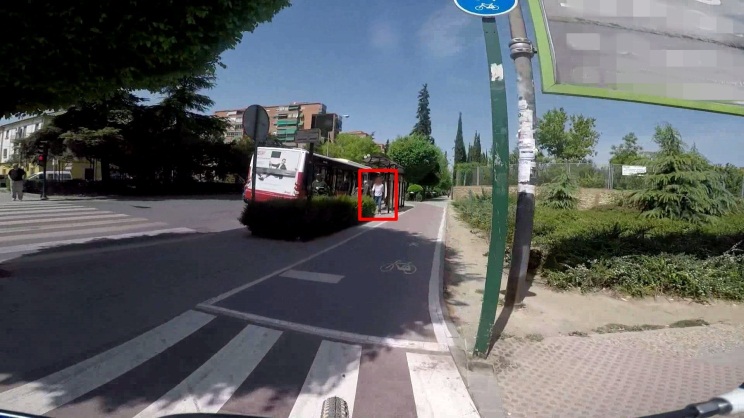 \| A pedestrian cross the cycle lane from the left \| \| Cycling_15 \| You are riding a bike downhill along a cycle lane, behind another cyclist. When you arriving to the cycle crossing, a vehicle driver does not brake his car to let you cross. The clip occludes just in the moment you realise he is not going to stop \| 14’ \| 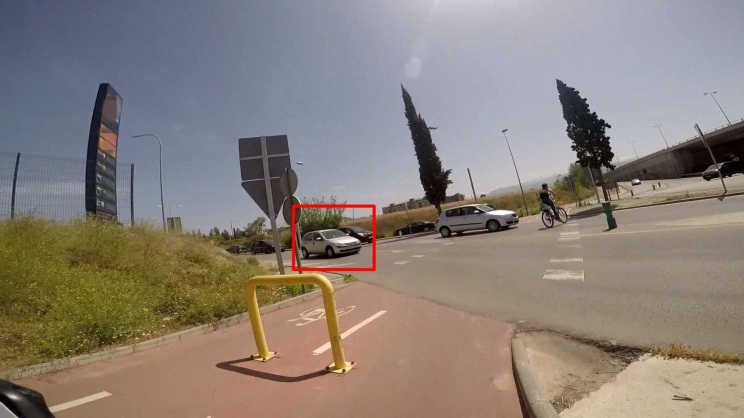 \| A car does not stop at a cycle crossing in a beltway \| \| Cycling_16 \| Two cars ahead of you are visible from the first moment of the clip. They are coming to a zebra crossing, and suddenly brake before cross it, because another car approaches to the intersection from the left. The clip occludes when both cars stop \| 12’ \| 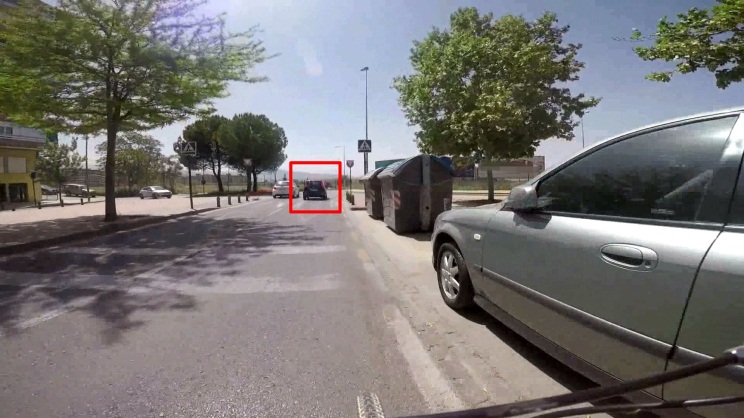 \| A car ahead of you stop just before the zebra crossing \| \| Cycling_17 \| Traveling on the skirts of the city, you ride a bike on the right shoulder of the road. In the distance, a person on horseback show up. The clip occludes at the point where the horse is totally perceptible \| 33’ \| 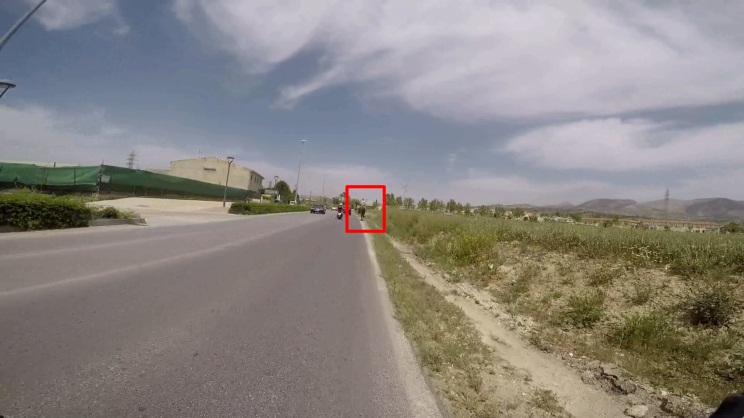 \| A person on horseback ahead of you is moving slowly forward \| \| Cycling_18 \| You are bicycling along a town road, when you observe a car being driven in the opposite direction. The clip occludes as you both are getting closer \| 10’ \| 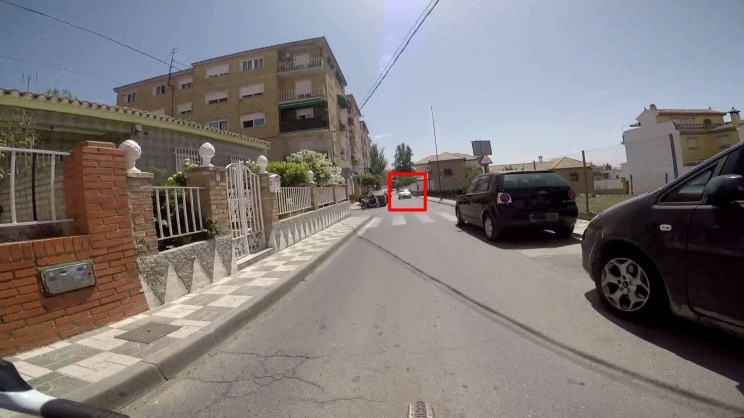 \| A car coming to you in the opposite direction \| \| Cycling_19 \| You are riding a bike around the town, when suddenly a motorcycle appears from your left side, coming from a big square. The clip occludes just in the moment which the motorcycle is perceptible \| 8’ \| 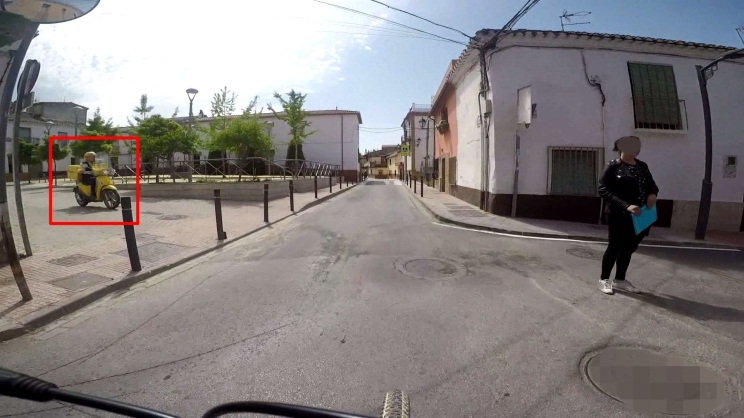 \| A motorcycle suddenly crosses the street from the left \| \| Cycling_20 \| Traveling on a town road, one pedestrian is walking at the left side of the road, and another one crosses the street from the right sidewalk. The clip occludes when the second one reaches the left sidewalk. \| 14’ \| 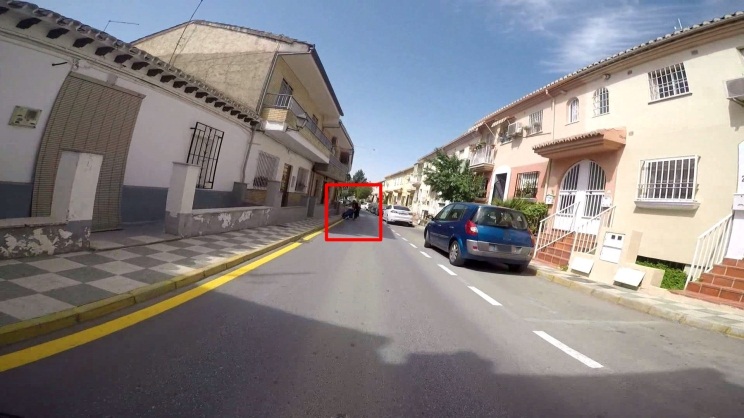 \| Two pedestrians walk across your lane \| |
| --- | --- | --- | --- | --- | --- | --- | --- | --- | --- | --- | --- | --- | --- | --- | --- | --- | --- | --- | --- | --- | --- | --- | --- | --- | --- | --- | --- | --- | --- | --- | --- | --- | --- | --- | --- | --- | --- | --- | --- | --- | --- | --- | --- | --- | --- | --- | --- | --- | --- | --- | --- | --- | --- | --- | --- | --- | --- | --- | --- | --- | --- | --- | --- | --- | --- | --- | --- | --- | --- | --- | --- | --- | --- | --- | --- | --- | --- | --- | --- | --- | --- | --- | --- | --- | --- | --- | --- | --- | --- | --- | --- | --- | --- | --- | --- | --- | --- | --- | --- | --- | --- | --- | --- | --- | --- |
