# Supplementary material for: Hazard Perception and Prediction test for walking, riding a bike and driving a car: “Understanding of the global traffic situation”
Source: PLoS One. 2020 Oct 16;15(10):e0238605. doi: 10.1371/journal.pone.0238605 (PMC7567349; doi:10.1371/journal.pone.0238605)
Supplement: S3 Table — A description of the hazards selected within vehicle traffic perspective’ clips. (DOCX) [file pone.0238605.s003.docx]

**S3 Table. Driving Clips**

A description of the hazards selected within vehicle traffic perspective’ clips

| Nº/Fig. | Driving clips | (sec.) | Last sketch,  prior the clip occlusion | Hazard |
| --- | --- | --- | --- | --- |
| Driving_1 | During driving across a narrow one-way urban road, a pedestrian is walking along the right side of the road. The clip occludes when, at the same time, a motorcycle comes into your lane, forcing you to brake | 31’ | 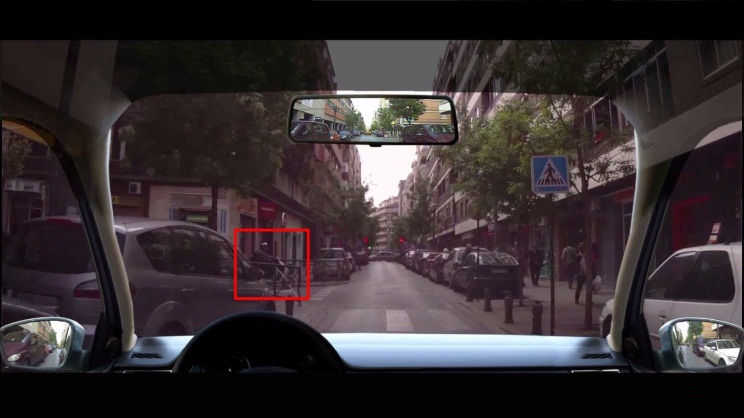 | A motorcycle swayed into our lane from the left |
| Driving_2 | You enter in a roundabout when it is safe gap traffic. The clip occludes just before your car is going to take the first exit, and there is a pedestrian walking along the right side there | 30’ | 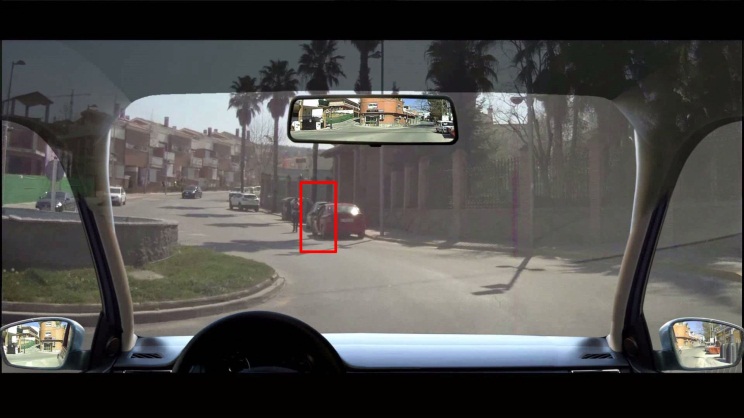 | A pedestrian is walking along the right side of our exit lane (in a roundabout). |
| Driving_3 | Turning right in an urban road, you suddenly find a motorcycle on your side of the road. The clip occludes when the motorcycle is starting to gets on the road | 24’ | 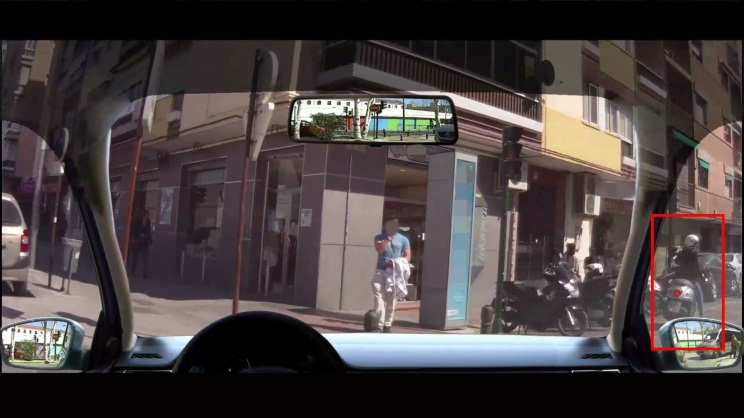 | A parked motorcycle from the right gets on the road |
| Driving_4 | A yellow car overtakes you from the right while a distracted pedestrian is crossing the street from the left. The clip occludes as the pedestrian moves towards the dashed white lines | 19’ | 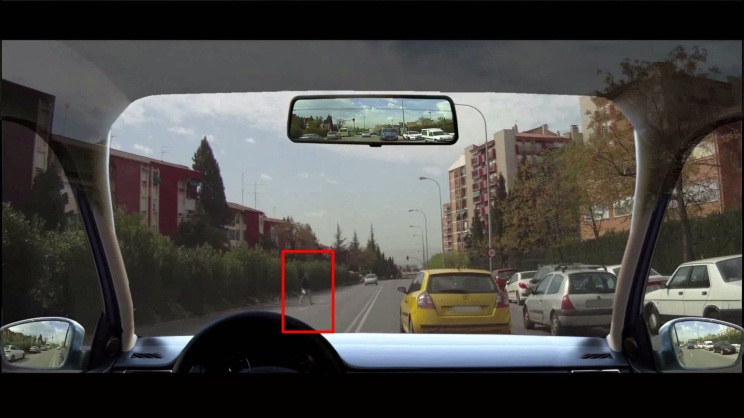 | A distracted pedestrian crosses the street from the left |
| Driving_5 | As you are driving, a car gets on your lane from the left. The clip occludes when the brake lights of this car switch on | 21’ | 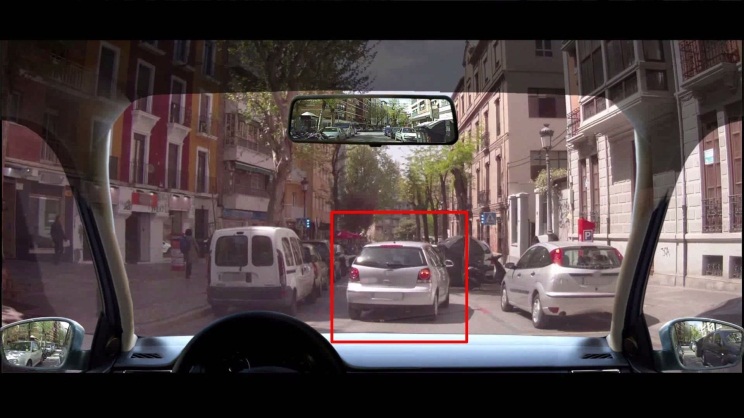 | A car ahead suddenly breaks |
| Driving_6 | Driving straight on an industrial park road, a car comes into your lane from the right, forcing you to break. The clip occludes just in the moment you can see the bonnet of the white car | 36’ | 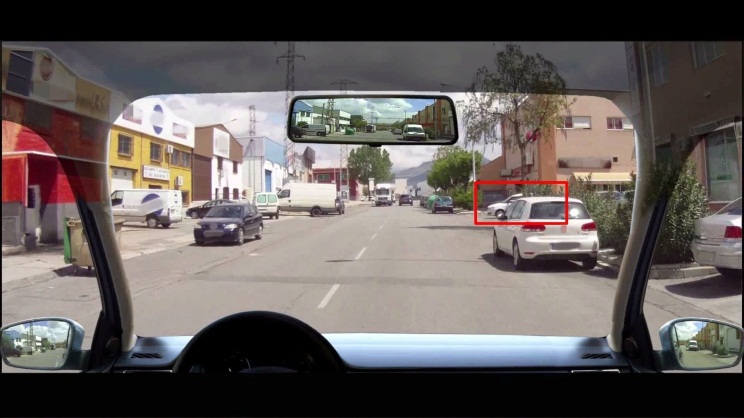 | A white car cross the lane we drive in from the right |
| Driving_7 | Driving around the vicinity of a school, you find a bottleneck. The clip occludes when a pedestrian is about to go through a zebra crossing | 18’ | 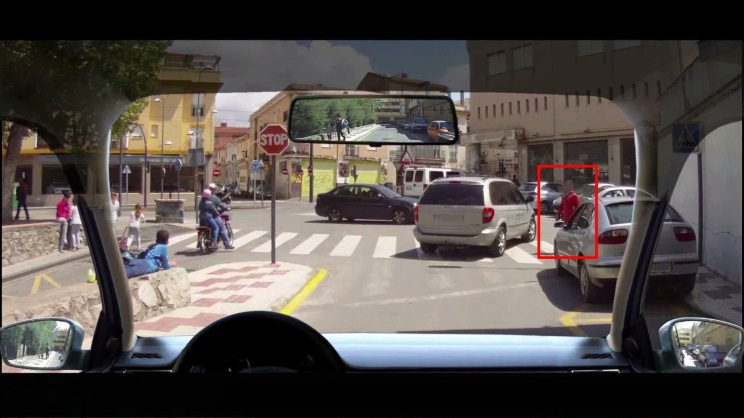 | A pedestrian cross the lane we driven in from the right |
| Driving_8 | Turning right while you are driving, you come across suddenly a bus that is getting into the lane you go to. The clip occludes when bus indicator starts to flash. | 34’ | 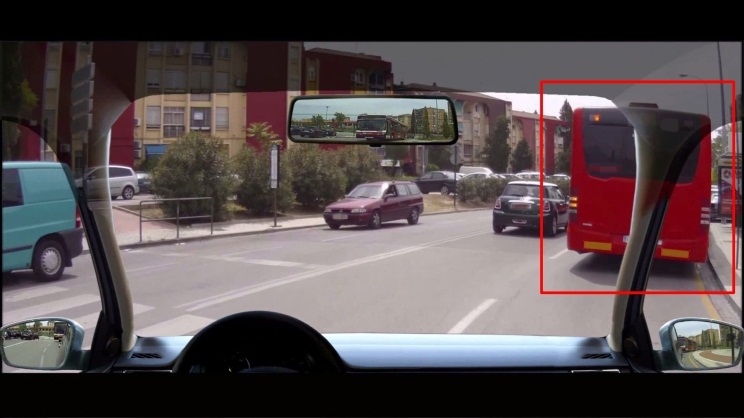 | A bus gets into the lane you go to |
| Driving_9 | Driving in an urban road, along the left lane, a motorcycle tries to overtake you from the right. The clip occludes when the motorcycle go from the right-side mirror to be directly visible | 19’ | 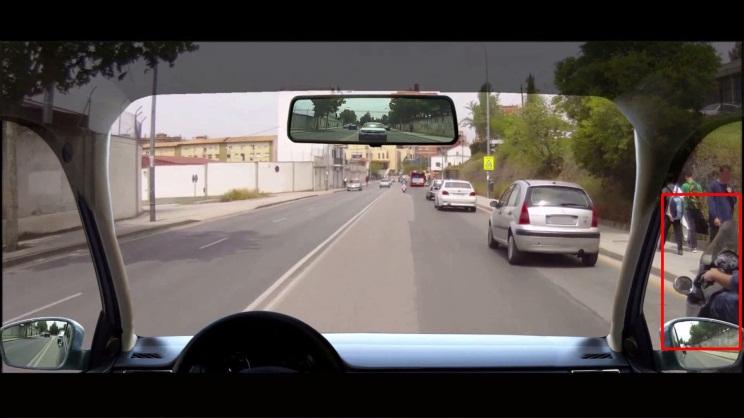 | A motorcycle that try to overtakes you from the right |
| Driving_10 | Getting into a two-way road, a cyclist is moving parallel to you, while a pedestrian crosses the street from the left. The clip occludes when the pedestrian is coming to the white line on the road. | 29’ | 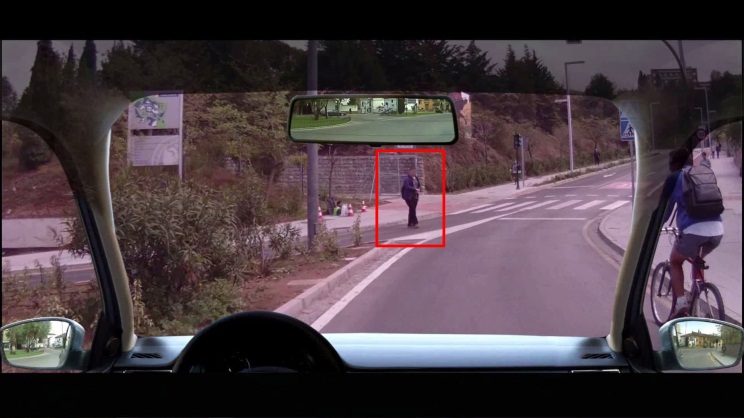 | A pedestrian crosses the street from the left |
| Driving_11 | Driving on a two-way urban road, a pedestrian start to cross the street from the left. The clip occludes when the body of the pedestrian can be seen completely | 9’ | 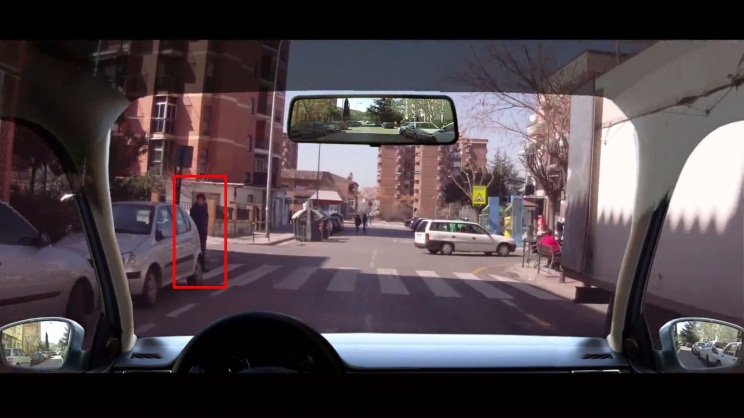 | A pedestrian crosses the street from the left |
| Driving_12 | Driving towards a car double-parked, a motorcycle gets into your lane suddenly. The clip occludes when the motorcycle is positioned ahead of you | 39’ | 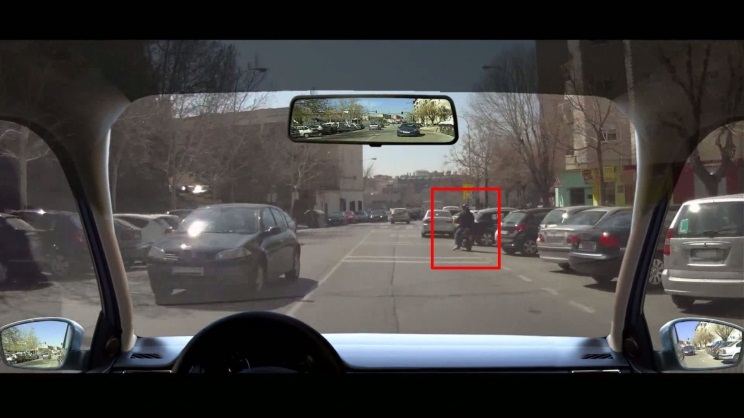 | A motorcycle crosses your lane from the right |
| Driving_13 | Traveling on a shopping center parking, you turn right and come across a car that is going into reverse from the left. The clip occludes when the boot is crossing your lane. | 28’ | 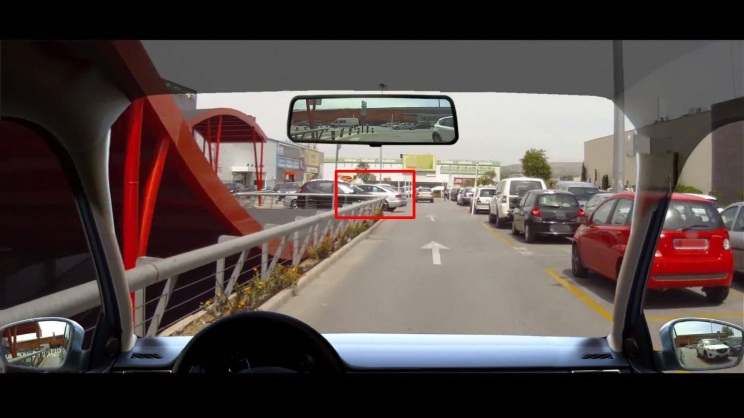 | A car gets into your lane in reverse way |
| Driving_14 | You are traveling on a unmarked single-lane road, on the outskirts of the city. A pedestrian is walking on the right side of the road. The clip occludes when you can see the body of the pedestrian completely. | 10’ | 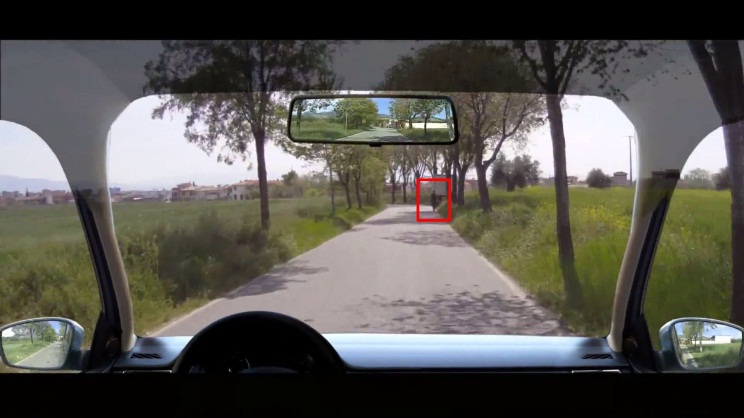 | A pedestrian who is walking on the right side of the road |
| Driving_15 | Driving along the highway, on the right lane, you travel towards the slip road. But you find out that there is a bottleneck there. The clip occludes when the bottleneck is perceptible | 12’ | 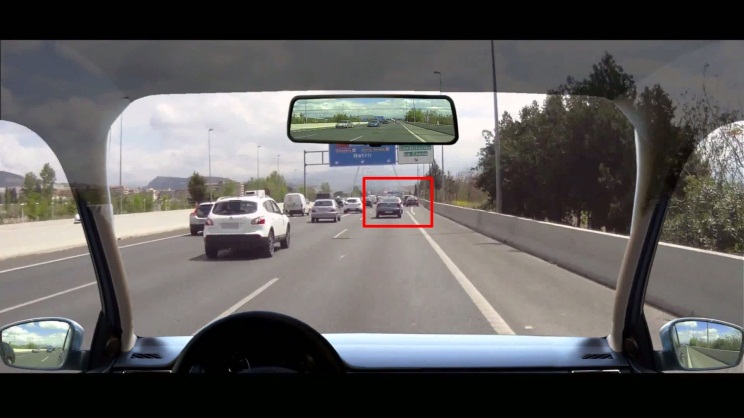 | A bottleneck in the slip road that you are going to |
| Driving_16 | You are driving on a two-way road, when you suddenly find out two maintenance service vehicles on the right side of the road. The clip occludes when the maintenance vehicles are visible | 25’ | 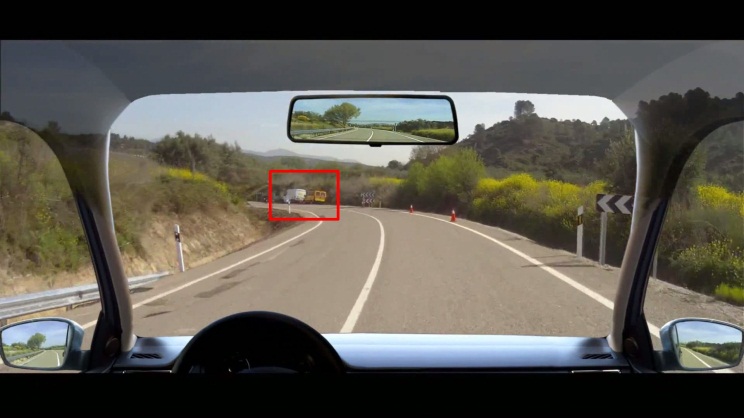 | Two maintenance service vehicles parked on the right side of the road |
| Driving_17 | Driving along an urban road, a pedestrian jumps and crosses the median strip from the left. The clip occludes just before he jumps again to your lane | 16’ | 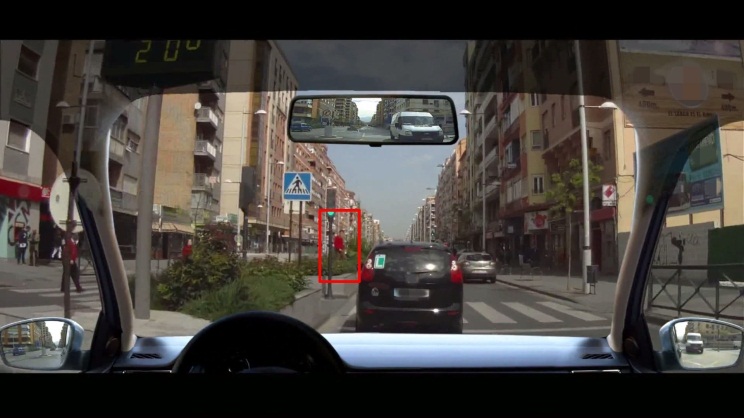 | A pedestrian crosses the median strip from the left side of the road |
| Driving_18 | You are driving along a narrow street. The co-pilot of a car leaves the vehicle when you are coming towards to her. The clip occludes when a previous car, behind her, allows you to see her | 35’ | 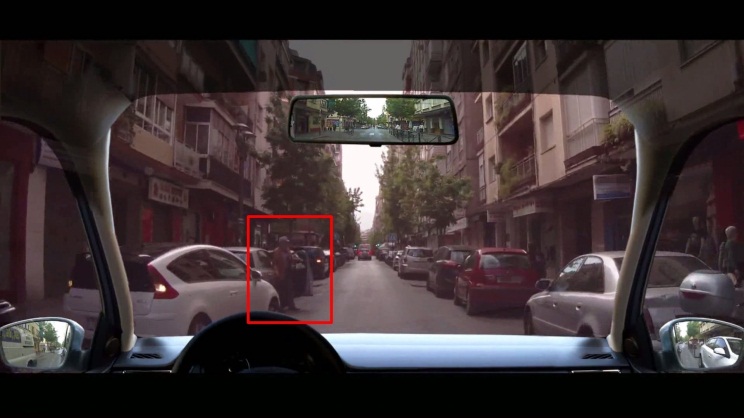 | A co-pilot who are leaving a car from the left side of the road |
| Driving_19 | Driving through an hospital area, an open car door is perceptible in the right side of the road. The clip occludes when a passenger is about to leave the car | 11’ | 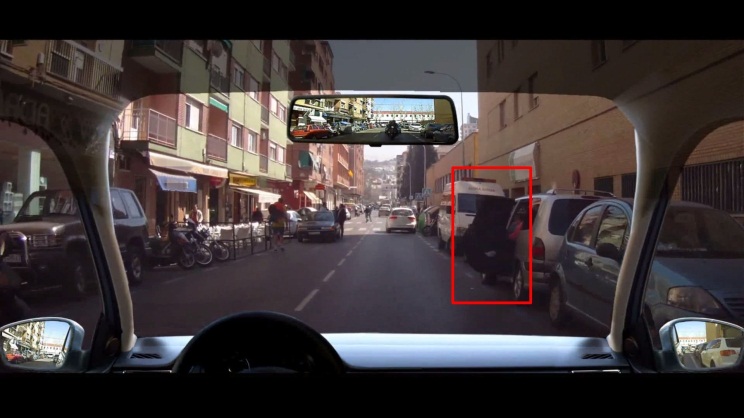 | A passenger who is leaving from a car at the right side of the road |
| Driving_20 | Traveling along a motorway, a lorry and a maintenance service vehicle are parked in the right shoulder of the road. The clip occludes when the maintenance vehicle looks to get into the road. | 16’ | 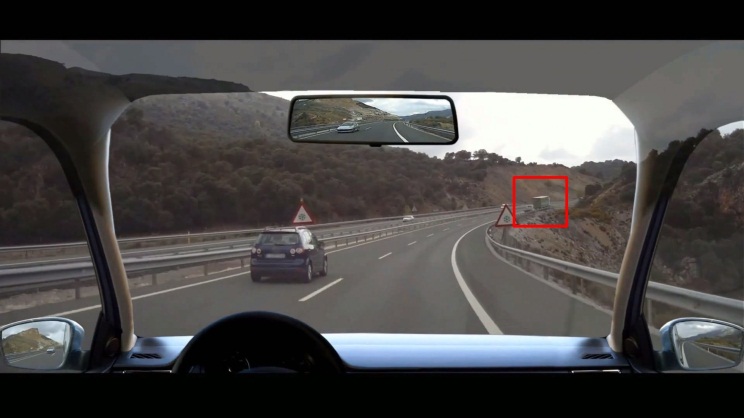 | Vehicles parked at the right shoulder of the motorway |
